# Virtual finger boosts three-dimensional imaging and microsurgery as well as terabyte volume image visualization and analysis

**DOI:** 10.1038/ncomms5342

**Published:** 2014-07-11

**Authors:** Hanchuan Peng, Jianyong Tang, Hang Xiao, Alessandro Bria, Jianlong Zhou, Victoria Butler, Zhi Zhou, Paloma T. Gonzalez-Bellido, Seung W. Oh, Jichao Chen, Ananya Mitra, Richard W. Tsien, Hongkui Zeng, Giorgio A. Ascoli, Giulio Iannello, Michael Hawrylycz, Eugene Myers, Fuhui Long

**Affiliations:** 1 Janelia Farm Research Campus, Howard Hughes Medical Institute, Ashburn, Virginia 20147, USA; 2 Allen Institute for Brain Science, 551 North 34th Street, Suite 200, Seattle, Washington 98103, USA; 3 Integrated Research Centre, University Campus Bio-Medico of Rome, 00128 Rome, Italy; 4 Department of Electrical and Information Engineering, University of Cassino and L.M., 03043 Cassino, Italy; 5 Department of Physiology, Development and Neuroscience, University of Cambridge, Cambridge CB2 3EG, UK; 6 Program in Sensory Physiology and Behavior, Marine Biological Laboratory, Woods Hole, Massachusetts 02543, USA; 7 Department of Pulmonary Medicine, M. D. Anderson Cancer Center, Houston, Texas 77030, USA; 8 Department of Biochemistry, Stanford University School of Medicine, Stanford, California 94305, USA; 9 Department of Molecular and Cellular Physiology, Stanford University School of Medicine, Stanford, California 94305, USA; 10 Circuit Therapeutics, Inc., Menlo Park, California 94025, USA; 11 New York University Institute of Neuroscience, New York University, New York, New York 10016, USA; 12 Krasnow Institute for Advanced Study, George Mason University, Fairfax, Virginia 22030, USA; 13 Max Planck Institute of Molecular Cell Biology and Genetics, Dresden, Germany; 14 BioImage, L.L.C., Bellevue, Washington 98005, USA; 15 These authors contributed equally to this work

## Abstract

Three-dimensional (3D) bioimaging, visualization and data analysis are in strong need of powerful 3D exploration techniques. We develop virtual finger (VF) to generate 3D curves, points and regions-of-interest in the 3D space of a volumetric image with a single finger operation, such as a computer mouse stroke, or click or zoom from the 2D-projection plane of an image as visualized with a computer. VF provides efficient methods for acquisition, visualization and analysis of 3D images for roundworm, fruitfly, dragonfly, mouse, rat and human. Specifically, VF enables instant 3D optical zoom-in imaging, 3D free-form optical microsurgery, and 3D visualization and annotation of terabytes of whole-brain image volumes. VF also leads to orders of magnitude better efficiency of automated 3D reconstruction of neurons and similar biostructures over our previous systems. We use VF to generate from images of 1,107 *Drosophila* GAL4 lines a projectome of a *Drosophila* brain.

## Abstract (web summary)

Large three-dimensional images are commonly generated through biological experimentation. Here the authors report software tools for exploration of three-dimensional images along with applications to assist in imaging, microsurgery, visualization and annotation of large image data sets.

## Graphical abstract

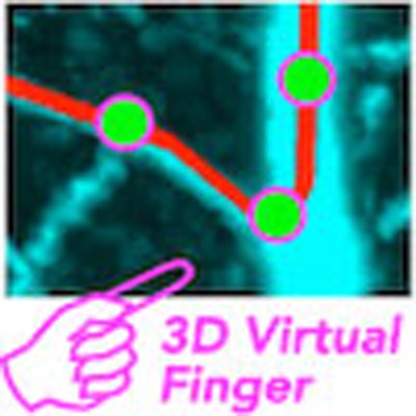

Microscopic imaging, bioimage data analysis and visualization are indispensable in many studies of modern high-throughput and quantitative biology^1^,^2^,^3^. A number of bioimage acquisition, visualization and analysis software packages, including public-domain tools such as ScanImage^4^, μManager^5^, MicroPilot^6^, ImageJ^7^, Vaa3D^8^, ilastik^9^, CellProfiler^10^, CellExplorer^11^, BrainExplorer^12^ and many commercial software suites such as Zen (Zeiss), Amira (VSG), Imaris (Bitplane), ImagePro (MediaCybernetics) and Neurolucida (MBF Bioscience), are being used widely. Despite a number of advances on visualization of multi-dimensional image data and automated analysis of such data (for example, automated mapping of a number of brain images to assemble three-dimensional (3D) brain maps^13^), a common bottleneck is the inability to efficiently explore the complicated 3D image content. This presents an obstacle for the unbiased, high-throughput and quantitative analysis of data and creates tremendous need for the development of new techniques that help explore 3D data directly and efficiently without expensive virtual reality devices. In addition to helping visualize, manage and annotate very large microscopic image data volumes, these new techniques may assist sophisticated analysis (for example, segmentation) of image data and various types of 3D-imaging experiments. These techniques may also be used in both pre-analysis procedures, such as image data acquisition and microsurgery, and post-analysis procedures, such as proofreading and editing of image analysis results.

Explicitly, ‘exploring 3D image content’ requires that a user is able to efficiently interact with and quantitatively profile the patterns of image objects using a graphical user interface of 3D image-visualization tools. The most widely used method to date is to scroll through cross-sectional slices of a 3D image stack, use a human–machine interaction device (for example, a computer mouse) to define objects of interests (for example, landmarks, cells and tissue regions) and thus profile these objects. This is essentially a two-dimensional (2D) method. For applications that involve large volumes or large numbers of images, this 2D process is not only time-consuming and low-throughput, but also brings bias to the understanding of intrinsic 3D properties of bioimage data^3^. Thus, inputting user-specified information of the observed image patterns through human–machine interaction becomes a major bottleneck of many quantitative biology applications, for example, proofreading and editing 3D-computed neuronal reconstructions from microscopy images^14^. Importantly, such prior information supplied by a user in real time could substantially improve the performance of automated image analyses^8^,^9^.

Overcoming this barrier calls for novel techniques that can map the identified 2D user input via 2D display devices, such as a computer screen, back to the 3D volumetric space of the image. Mathematically, this is of course a difficult inverse problem. Previously, we published a method embedded in the interface of Vaa3D that allows pinpointing a 3D location in the image volumetric space with only one or two computer mouse clicks^8^. Similar approaches were also adopted recently in both public-domain non-profit projects (for example, Janelia FlyAnnotation WorkStation for selection of colour-separated neurons) and commercial systems (for example, Neurolucida (MBF Bioscience) for selection of 3D-imaged neuronal spines). This approach has also been extended to create curves. For instance, by manually or automatically concatenating a series of pinpointed 3D locations, one could generate a simple 3D curve with Vaa3D. Alternatively, using the Imaris (Bitplane) software, a user may produce a 3D curve by first defining a parameterized starting location followed by region growing or tube-fitting.

Unfortunately, all of the above 3D interaction methods are still very burdensome and prone to error for complicated image content and large data sets. Here we introduce a family of new Open Source computing methods called 3D virtual finger (VF). The VF methods generate 3D points, curves and regions of interest (ROI) objects in a robust and efficient way. As long as these objects are visible in 2D display devices, one mouse click (or an equivalent operation of other similar input devices such as a digitizer pen or a touch screen) allows VF methods to reproduce their 3D locations in the image volume.

The VF technology allows instant and random-order exploration of complex 3D image content, just like our real fingers explore the real 3D world using a single click or stroke to locate 3D objects. Here we report several technologies in imaging and image-related procedures, including image data acquisition, visualization, management, annotation, analysis and the use of the image data for real-time experiments such as microsurgery, which can be boosted by 3D-VF. In particular, we highlight three case studies: (1) instant 3D imaging, profiling and free-form *in vivo* microsurgery of any 3D ROI in experiments with *Caenorhabditis elegans* and *Drosophila*, (2) instant 3D visualization and annotation of terabytes of microscopic image volume data of whole-mouse brains and (3) efficient reconstruction of complicated neuron morphology from images of dragonfly and other systems containing very high levels of noise, enabling the generation of a projectome of a *Drosophila* brain.

## Results

### 3D virtual finger

**Figure 1:**
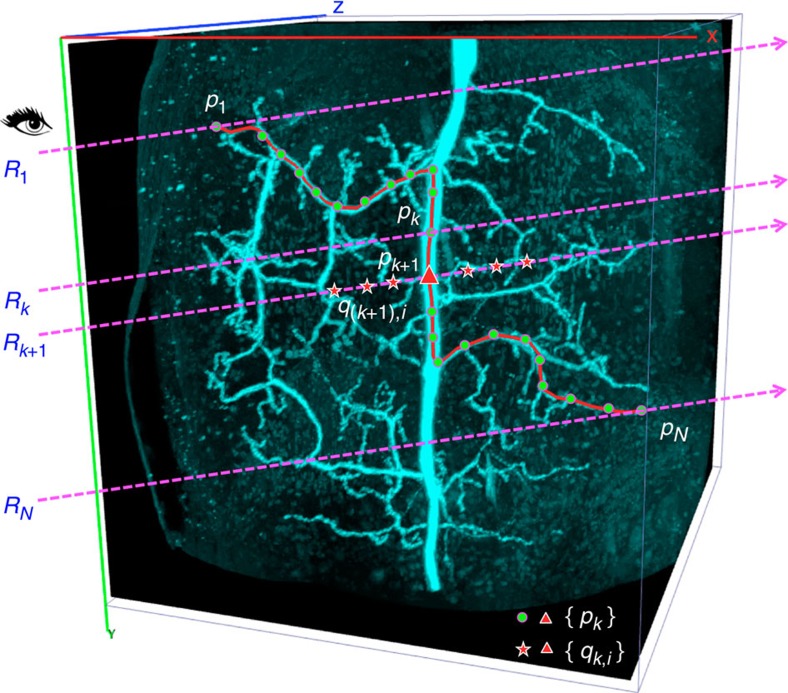
Curve drawing methods in the family of 3D VF algorithms. CDA1 and CDA2 for generating a 3D curve using one computer-mouse stroke painted in the 2D projection of a 3D image of a dragonfly thoracic ganglion neuron. *R*_1_~*R*_N_: the first to the last shooting rays, which are parallel to each other and along the path of the mouse stroke. *p*_1_~*p*_N_: the estimated 3D location of each curve knot, each corresponding to a shooting ray. *q*_*k*,*i*_ and *q*_(*k+*1),*i*_: the one-voxel evenly spaced 3D locations along the *k*th and (*k*+1)th rays, respectively; the final knot location *p*_*k*_ for the ray *R*_*k*_ is selected from the set.

**Figure 2:**
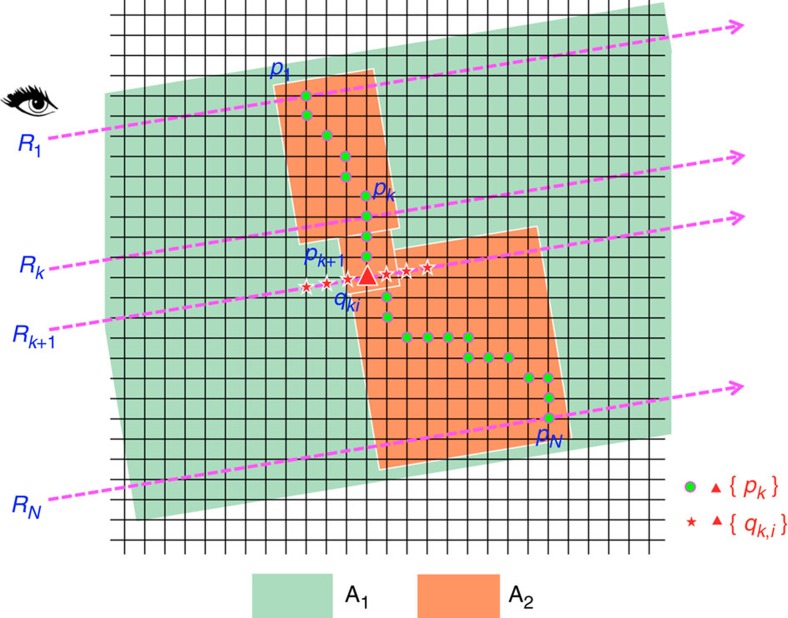
Schematic illustration of several different methods of CDA. Case 1: *p*_1_ is determined using PPA, then search *p*_2_ on the ray *R*_2_ within a small range (the default was set to ±30 voxels in our software) around the location of *p*_1_. Once *p*_*k*_ is found, the same method is reused to find *p*_*k*+1_. This scheme is the CDA1 method, which is fast and useful for drawing in dark region, but is sensitive to the starting location. Case 2: Instead of determining *p*_1_ using PPA, we directly use fast-marching to find the shortest Geodesic path between all possible points on the rays *R*_1_ and *R*_2_. The hit points will be called *p*_1_ and *p*_2_. Next, we find the shortest path between *p*_2_ and the ray *R*_3_, and thus find *p*_3_. This process is repeated until all rays have been searched. This is the basic CDA2 method. Note, as all possible combination paths between *R*_1_ and *R*_2_ have been searched, this method is not sensitive to noise or obscuring of 3D objects (Supplementary Movies 6 and 7). Case 3: In CDA2, instead of finding the shortest path between one single hit point *p*_*k*_ on the ray *R*_*k*_ to the next ray *R*_*k*+1_, we find the shortest paths for all consecutive rays. This then allows us to compute and choose the global minimum cost path starting from the first ray and ending at the last ray, in all possible combinations of initial paths through consecutive rays. The entire search area A1, that is, the whole overlapping area of rays and the 3D image, is used. This is called the globally optimal CDA2 method (Supplementary Movie 5). Case 4: We can first use PPA to determine preliminary hit points on a pair of consecutive rays, based on which a smaller search area *A*_2_ is determined. *A*_2_ consists of a series of margin-extended and tilted bounding boxes (default margin is 5 voxels). Next, we can restrict the search of CDA2 on *A*_2_, instead of a much bigger region *A*_1_. This scheme is called the bounding-box-restricted CDA2. Of note, for all above cases (and other additional cases explained in the Methods), we restrict the search to voxels only (instead of sub-voxel locations).

**Figure 3:**
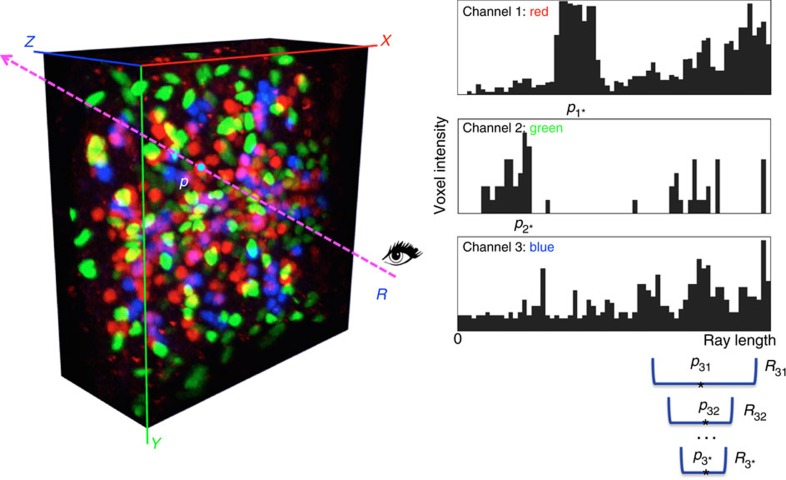
PPA_*c*_ for multiple colour-channel 3D pinpointing using one computer-mouse click. Image shown: a confocal image of a *Drosophila* late embryo where cells are labelled using fluorophores with different colours. *R*: a shooting ray from the observer to the on-screen 2D mouse-click locus. *p*: the final 3D location estimated by finding the one with the maximal intensity among candidates *p*_1*_, *p*_2*_ and *p*_3*_, which are detected for all colour channels independently. For each colour channel, the progressive mean-shift method is used to narrow down the search range *R*_*ik*_ (in this case *i*=1, 2, 3, and the iteration indicator *k*=1, 2, …) along the shooting ray until convergence.

VF includes a family of *3D-WYSIWYG* (‘what you see in 2D is what you get in 3D’) computer algorithms that map users’ inputs in the 2D plane of a computer screen to the 3D locations of biological entities (for example, cells, neurons or microtubules) in the volumetric space of a 3D image stack (Figs 1, 2, 3). Here we have focused on producing 3D points, curves and ROI objects in a 3D image with a single stroke or click of computer mouse. This single action is performed on the 2D maximum intensity projection of the 3D volumetric image by a monitor. The three types of objects produced with VF correspond to important structures found in typical fluorescent microscopic images: 3D points may mark locations of labelled cells or proteins, 3D curves may correspond to general vessel-like structures, such as microtubules, blood vessels, bronchial trees or neurite processes, and 3D ROIs may highlight specific cell populations or brain compartments.

A 3D point-pinpointing algorithm (PPA) (Fig. 3) uses one mouse click to determine the location of a point in a 3D image volume as long as there is a visible object at this location. Our new PPA methods supersede the limitations of an original method^8^, which was neither directly applicable to multi-channel images nor robust for noisy images. Here we have developed a novel PPA, called PPA_*c*_, which works effectively for multi-channel (for example, multiple fluorescent colours) 3D images (Fig. 3 and Supplementary Movie 1). We further extended PPA_*c*_ to a very robust variant called PPA_*n*_ for images that are contaminated by strong noise. This was attained by using a very short mouse stroke around the mouse-click location, based on the curve-generation function curve-drawing algorithm 2 (CDA2) described below (Supplementary Movie 2).

The most important algorithms in VF are two newly designed 3D CDA, namely CDA1 and CDA2, based on a single stroke of a computer mouse (Figs 1 and 2, and Supplementary Movie 3). Both methods use the trajectory of cursor locations on a 2D screen sampled from a mouse stroke. Through each cursor location, we generate a shooting ray orthogonal to the screen. In CDA1, we use PPA_*c*_ to estimate the 3D location of the first knot on the curve. Next, we iteratively estimate the 3D location of the next knot on the curve by applying PPA_*c*_ to the subsequent ray, but within a small neighbourhood range around the last determined knot. CDA1 is fast. However, when the mouse stroke deviates from a bright image object due to the shaking of a user’s hand or occlusion of several objects, CDA1 might not be robust enough to converge to that bright object. Although this sensitivity is actually useful for producing a 3D ROI in the dark area of an image (see below), it can be a drawback in other situations. Thus, we derived CDA2, which makes curve generation prone to bright signals. CDA2 uses an adapted fast-marching algorithm to search a shortest geodesic path from the last knot to the current ray (Methods). CDA2 iterates this process to complete the entire curve. We also provide additional flexibility by designing methods to refine a 3D curve using extra mouse operations (Methods).

**Figure 4:**
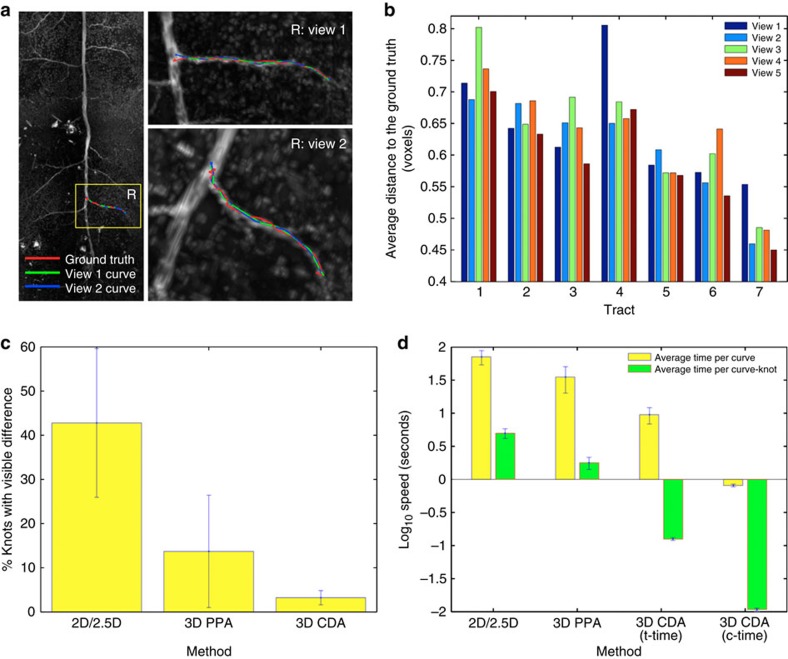
Evaluation of CDA. (**a**) CDA generates consistent 3D neurite tracts (curves) (green and blue) that are very close to the ground truth (red) regardless of different viewing angles. Image: 3D confocal image of a heavy-noise-contaminated dragonfly thoracic ganglion neuron. The ‘ground–truth’ curves were generated using Vaa3D-Neuron1 (ref. 8) and were also manually inspected to ensure that they are correct. (**b**) Distances between the 3D neurite tracts (curves), which are generated from different angles and different zooms, and the ground truth. Data is based on 1,470 measurements of 7 tracts in the image in **a**. (**c**) Percentages of curve knots that have visible spatial difference (≥ 2 voxels) (mean±s.d.). 2D/2.5D: manual generation of a 3D curve based on first mouse-clicking on 2D cross-sectional XY planes in a 3D image, or using all three XY, YZ and ZX cross-sectional planes (2.5D), and then concatenating these locations sequentially. 3D PPA: manual generation of a 3D curve based on first mouse-clicking in the 3D-rendered image using PPA to produce a series of 3D locations, and then concatenating them. Data are based on tracing the primary projection tracts in five 3D dragonfly confocal images where the curve generation is possible for all the 2D/2.5D, 3D PPA and 3D CDA methods. (**d**) Speed of 3D curve generation using different methods (mean±s.d.). c-time, computing time for CDA; t-time, total time (including human-machine interaction and c-time) for CDA. Image data are the same in **c**.

CDA2 is robust in generating consistent and precise 3D curves from different viewing angles, zooms and other perspective parameters of an image (Fig. 4a and Supplementary Movies 3, 4, 5, 6, 7). To analyse an image contaminated with heavy noise, we used CDA2 to generate 1,470 measurements for the 3D coordinates of 294 knots of 7 curves. We found that the average spatial divergence (SD) of these curves is ~0.65 voxels from the respective ‘optimal ground-truth’ curves (Fig. 4b) (Methods). On average, only 1.61% of these measured locations have visible SD (≥2 voxels) (Supplementary Fig. 1a), while the maximal spatial separation is around 2.09±0.95 voxels (Supplementary Fig. 1b). Compared with two alternative approaches that generate a 3D curve based on a series of mouse clicks on 2D planes or in the 3D space directly using PPA, CDA2 improves the accuracy ~13 and 4 times, respectively, for clearly visible tracts (Fig. 4c). For tracts with weak signals, the other two methods fail, while CDA2 still works well (for example, the fourth tract in Supplementary Movie 3).

CDA2 is very fast and is suitable for real-time 3D operations. Although the human–computer interaction time of a single mouse stroke usually varies between 5 to 20 s, CDA2 takes on average ~500 ms per curve on a 1.8-GHz MacBook Air laptop computer. Compared with the other two methods, CDA2 is ~40 times faster for generating each curve knot (Fig. 4d). The total combined time of CDA2 and the human–machine interaction is still 7.5 times faster (Fig. 4d). CDA1 is even faster, and typically just takes 1~5 ms to generate a curve.

We also produced a 3D ROI using one mouse click or stroke or zoom-in operation(s) (Supplementary Movie 8). In the one-click case, we first called PPA_*c*_ or PPA_*n*_ to determine a 3D location. Next, the ROI is defined as a size-adjustable, cube-shaped local window, normally with 33^3^ voxels, surrounding this specific location. In the one-stroke case, the bounding box of a 3D curve generated using CDA1 or CDA2 is defined as the ROI. In the one zoom-in operation, the four corners of the viewport of the 3D-rendered image are used to generate four rays, to which the PPA_*c*_ is applied to produce a 3D ROI. To obtain such 3D ROI with a zoom-in operation, users can also choose between other similar but alternative algorithms that we developed in our software system.

### Instant 3D imaging and 3D free-form microsurgery

Although great advances have been made in recent years to improve both the speed and resolution of optical microimaging^15^,^16^,^17^,^18^, very little work has been done in directing the imaging system to acquire data and perform experiments only at 3D locations that are relevant and necessary. This is partially due to the design of optical imaging systems (for example, selective plane illumination^18^) and hardware pieces (for example, resonant scanning mirror and fast cameras) that favour the ‘lump-sum’ imaging. Yet, a more important reason is that it is very challenging to identify the 3D ROIs in real-time experiments. We applied VF to 3D laser scanning imaging, one of the most widely used optical imaging techniques, to demonstrate the improvement in the imaging speed, flexibility and capability to do previously difficult experiments.

We tested VF on our SmartScope system, which is a custom-built laser scanning microscope with a specially designed controlling module. The module is based on semi-automated and fully automated on-board image analysis functions that enable smart control of the laser scanning microscope. We designed a Vaa3D plugin, SmartScope-controller, to connect SmartScope and the Vaa3D system^8^, on top of which we added the VF functions.

**Figure 5:**
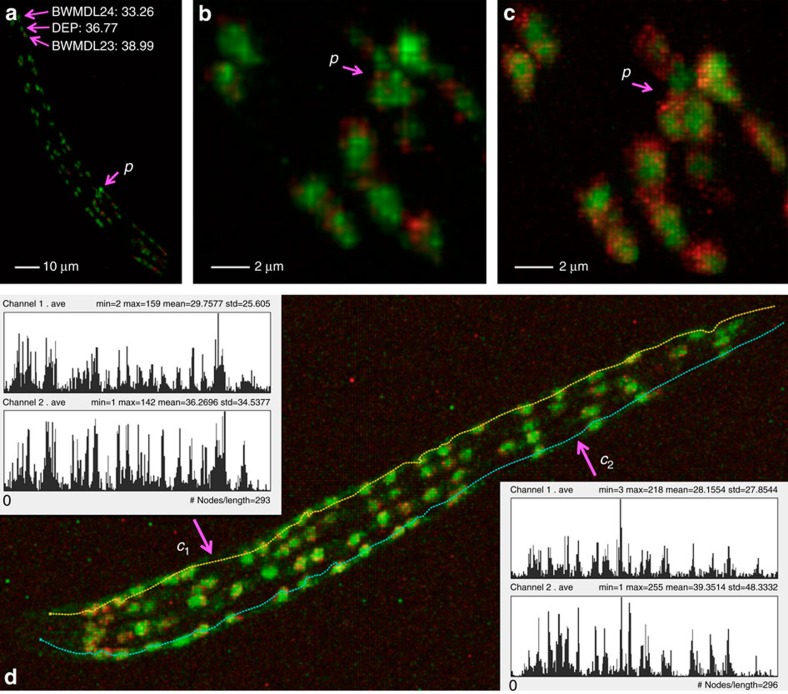
Instant 3D zoom-in imaging and quantitative measurement of single-nucleus gene expression for *C. elegans*. (**a**) A 3D pre-scan image of L1 *C. elegans*, along with the instant 3D measurements of gene expression levels of three cells BWMDL23, DEP and BWMDL24. *p*: location of interest for zoom-in imaging at a higher resolution. (**b**) Digital zoom-in of the area around *p*, where the insufficient voxel resolution does not manifest clear nuclei boundary. (**c**) Optical zoom-in around *p*, where the boundary between several nuclei is visible. (**d**) Instant 3D measurement of the gene expression of two bundles of body wall muscle cells, without computational segmentation of cells. For the profile of each curve of *c*_1_ and *c*_2_, the top is the gene expression of channel 1 (red) and bottom is that for channel 2 (green). Red: Punc54::H1::cherry. Green: Pmyo3::eGFP.

VF allows instant imaging of any location in the specimen without the need to predefine an ROI based on 2D planes, which is the prevailing method employed by various imaging systems. In a typical experiment with VF, we first scanned a low-resolution 2D or 3D image *I*_*Pre*_ (Fig. 5a and Supplementary Fig. 2a). Next, we use one computer-mouse click or stroke to define an ROI of *I*_*Pre*_in real time (Fig. 5a and Supplementary Fig. 2a), for which a higher resolution 3D image is instantly scanned (Fig. 5c and Supplementary Fig. 2c). This ‘instant optical zoom-in’ acquisition approach produces much sharper images than simple digital zoom (Fig. 5b and Supplementary Fig. 2b). In this way, we eliminated the need to scan a large area of the specimen in high resolution, which, in many cases, contains only a very small fraction of useful signal (often at the range of 0.1% of the entire volume of a specimen). Without changing the design or hardware of an optical imaging system, scanning such small areas based on adaptively defined ROIs is hundreds of times faster than scanning the entire specimen.

Further, we can quantitatively profile in real-time any ROI (for example, cell) that is visible in *I*_*Pre*_. For instance, we measured single-cell resolution gene expression of three cells in live L1 stage *C. elegans* animals within 10 s (Fig. 5a). To profile the gene expression level for an entire, but curved, bundle of body wall muscle cells (Fig. 5d), we only used one mouse stroke, which took a few seconds at most, to determine the skeleton curve of such a bundle of cells. In contrast, generating a similar measurement in a previous study^19^ required ~2 h of image acquisition in a chemically fixed *C. elegans* sample, followed by the application of a sophisticated image analysis method offline^11^.

**Figure 6:**
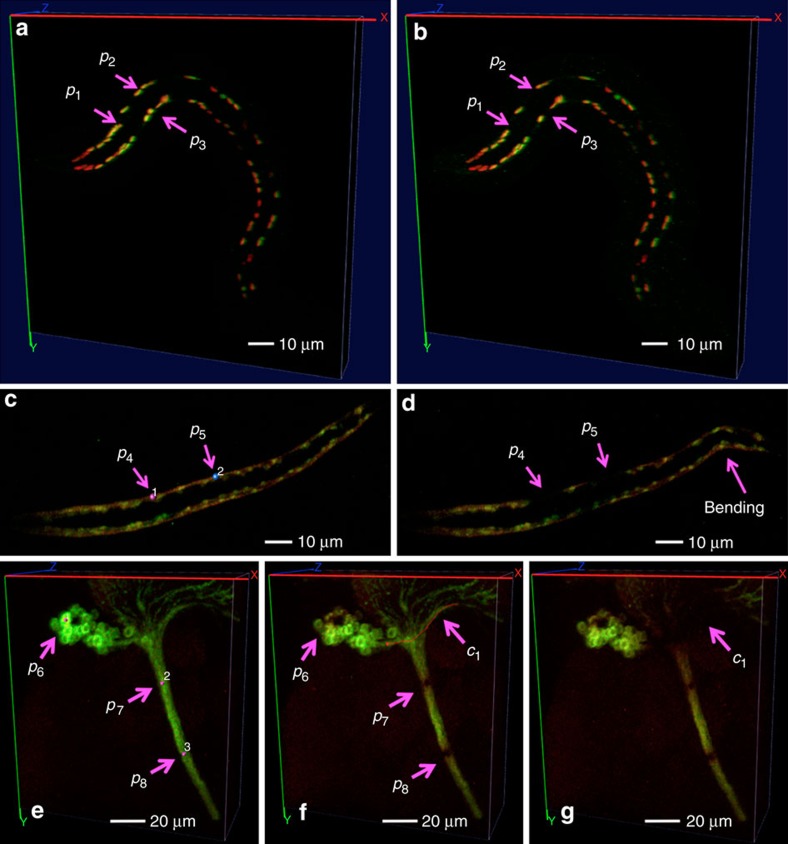
Instant 3D microsurgery for different animals. (**a**) Instant 3D pinpointing of body wall muscle cells in a fixed L1 *C. elegans* worm. *p*_1_, *p*_2_, *p*_3_: three muscle-cell nuclei. Red: Punc-54::H1::mCherry. Green: Pmyo-3::eGFP. (**b**) Instant 3D bleaching of the muscle cell nuclei in **a**. (**c**) Instant 3D pinpointing of muscle cells in a live L1 *C. elegans* worm. *p*_4_, *p*_5_: two muscle cells. Red: Pmyo-3::tagRFP-T. Green: Pmyo-3::GCaMP3. (**d**) Instant 3D bleaching of the muscle cells in **c** leads to bending of the animal. (**e**) Instant 3D pinpointing for an ato-GAL4-labelled *Drosophila* brain. *p*_6_: a cell body of a neuron. *p*_7_, *p*_8_: two loci on a major ato-GAL4 neurite tract. Green: ato-GAL4 pattern. (**f**) Instant 3D bleaching of locations in **e** and instant 3D curving for the same specimen. *c*_1_: a 3D curve cutting through the arbor of ato-GAL4 pattern in the optic lobe. (**g**) Instant 3D bleaching of the *c*_1_ curve in **f**.

Next, we used VF to control a 100-mW laser in the SmartScope system, in a point-and-shoot way, to instantly bleach or ablate multiple spots in fluorescently labelled specimens with accurate 3D localization. For fixed L1 stage *C. elegans* worms where a nucleus has a diameter of ~2 μm, we bleached single nuclei without affecting immediate neighbouring nuclei (Fig. 6a,b). This indicates that the precision of our system is within 2 μm and thus is appropriate for single-cell resolution bleaching and microsurgery experiments. We further applied this system to immobilized live L1 *C. elegans* larvae. Shooting muscle cells directly in 3D (Fig. 6c) instantly bleached the fluorescent signals at these locations (Fig. 6d) and at the same time caused the animals to bend substantially (Fig. 6d).

Finally, we used CDA2 to drive the bleaching laser to ‘cut’ a specimen instantly after we created a 3D curve in the pre-scanned image. We applied this 3D ‘optical surgical knife’ to complex structures. For example, in an ato-GAL4-labelled *Drosophila* brain, we bleached the neurite locally and precisely (Fig. 6e–g). As the 3D curve generation has sub-voxel accuracy (Fig. 4), the precision of this free-form optical knife is only limited by the actual setting of the optical imaging system used.

### Instant 3D visualization and annotation of terabytes of image volumes

New imaging technologies such as selective plane illumination microscopy (SPIM)^18^,^20^,^21^,^22^,^23^ and resonant scanning microscopy^24^ offer a fast speed, high resolution, a broad area and a long duration of imaging, as well as the ability to collect multiple data channels, each of which is associated with certain labelled bio-entities (for example, proteins or cells). In these situations, the volume of a typical 3D image stack easily surpasses the gigabytes range. Indeed, currently an important frontier of the field is how to handle terabytes of image volume smoothly. However, such Big-Image-Data poses substantial challenges for current computer hardware and software platforms. Two notable technical barriers are how to visualize and annotate such massive multi-dimensional data sets efficiently^14^.

**Figure 7:**
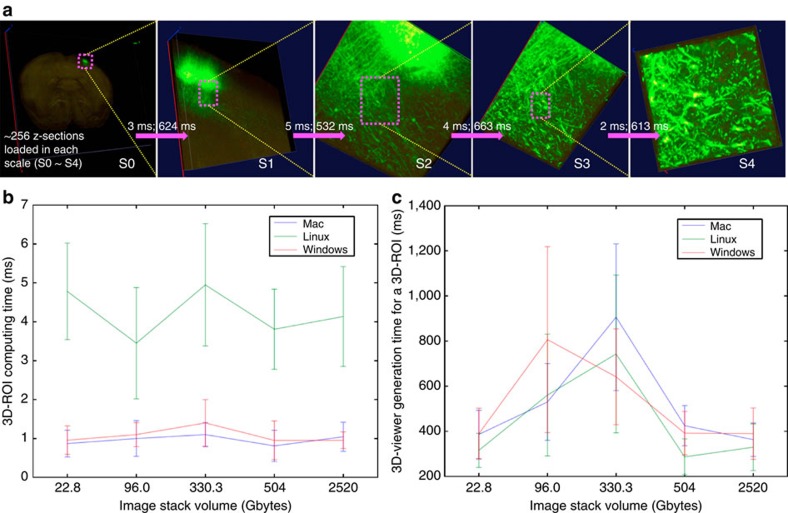
Instant 3D visualization of massive 3D image data stacks. (**a**) Visualization of a 2.52-Tb whole-mouse brain image stack, which has 30,000 × 40,000 × 700 voxels and three 8-bit fluorescent channels. For each scale (S0~S4) when a user is using 3D VF’s one mouse stroke feature to zoom-in at arbitrarily defined 3D ROI, the ROI-computing time and the actual 3D rendering time for this ROI are also shown on top of each magenta arrow. (**b**) Bench-test of ROI-computing time (mean±s.d.) for five different large image stacks of different properties on different operating systems (Mac, Linux and Windows). The five images are: single mouse neuron (22.8 Gb, 16 bit, single channel, 11.4 Gigavoxels), two rat neurons (96.0 Gb, 8 bit, two channels, 48.0 Gigavoxels), hippocampus (330.3 Gb, 8 bit, single channel, 330.3 Gigavoxels), whole-mouse brain (504 Gb, 8 bit, 3 channels, 168 Gigavoxels) and another whole-mouse brain (2.52 Tb, 8 bit, 3 channels, 840 Gigavoxels). See Methods for the configurations of machines used in these tests. (**c**) The actual 3D rendering time to visualize the image contents in each computed ROI, bench tested for the same data sets in **b** and various operating systems.

To demonstrate that VF can boost visualization of very large image volumes in a 3D dive-in way, similar to how Google Earth allows viewing satellite imageries, we developed a new Vaa3D-TeraFly plugin (Supplementary Movies 9, 10, 11) on top of the Vaa3D system. As VF allows a user to instantly identify an ROI in a 3D visualized image volume using a single finger operation, Vaa3D-TeraFly directly loads and renders the volumetric data corresponding to this ROI at a higher resolution (Fig. 7a and Supplementary Movies 9 and 10). This process maximizes the speed of data reading. Using regular computers and operating systems (Mac, Linux and Windows), we tested visualization of 3D image volumes with the sizes 22 Gb, 96 Gb, 300 Gb, 600 Gb and 2.5 Tb (entire mouse brain). In this case, the ROI computing was done mainly using CDA1. The computing time for a 3D ROI is only 1~7 ms (Fig. 7b), whereas the total response time of data loading and 3D rendering is well within the sub-second range on average (Fig. 7c and Supplementary Movie 10). Remarkably, both the ROI computing time and total time for 3D visualization of an ROI were almost constant, regardless of the overall size of the data sets tested, demonstrating that VF can be applied to unlimitedly large image stack.

VF also allows real-time 3D annotation of complex objects in images of massive size (Supplementary Movie 11). This feature has also been incorporated in Vaa3D-TeraFly software to count cells and label neurons or filaments quickly (Supplementary Movie 11). In our use cases, for example, precise profiling of cell counts in various brain regions, cells can be segmented offline and then imported to be quickly proof edited using Vaa3D-TeraFly. Another example is that the ability to quickly change the visualization resolution for terabytes of volumetric data, as well as to easily move from one region to an adjacent one at high-resolution, speed-ups proof editing of: (i) large tree-like structures of different sizes such as the vascular system, (ii) long thin structures such as axons.

Although the above 3D method substantially increases the efficiency when annotating massive data sets, this step is still a manual operation. To make this analysis and annotation processes easier, we have developed a new automation method as explained below based on VF.

### Fast neuron and projectome reconstruction

Digital reconstruction (tracing) of neuron morphology and circuits from 3D light microscope images is critical for understanding how a brain works^25^. Yet, due to the high complexity of neuron morphology and heavy noise in an image, it remains difficult to achieve 3D reconstructions of neurons quickly and reliably^26^. In such complicated image analysis situations, there are normally two processing steps: generation and proof editing of a reconstruction. Here we demonstrate the advantages of VF in both steps by using it as part of a newly developed Open Source software package, Vaa3D-Neuron2.

**Figure 8:**
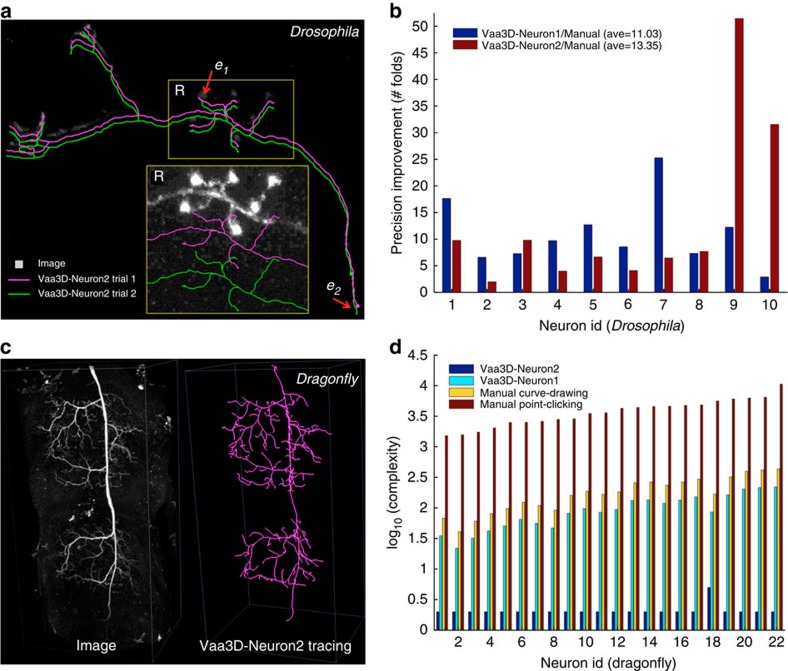
Neuron reconstruction using Vaa3D-Neuron2. (**a**) Reconstructions of a *Drosophila* projection neuron from a dark 3D confocal image. In the zoom-in region *R*, the image intensity is enhanced for better visibility. The two reconstructions are produced using two different tracing spans defined by two independent sets of landmark points. The reconstructions are intentionally offset from the image for comparison. *e*_1_ and *e*_2_: locations of small discrepancy of the two reconstructions. (**b**) Improvement of the reconstruction precision for ten *Drosophila* neurons, each with two independent trials of reconstructions. Manual reconstructions here were produced using Neurolucida. (**c**) Reconstruction of a densely arborized dragonfly thoracic neuron from a 3D confocal image with heavy noise. (**d**) Comparison of the computational complexity of competing methods for faithful reconstruction of 22 noise-contaminated dragonfly neurons.

In the reconstruction–generation step, we primarily considered the challenging scenario posed by an image with a low signal-to-noise (SNR) ratio. In this case, Vaa3D-Neuron2 often uses two distal 3D landmark points, each of which is produced using one mouse operation of PPA, to define a rough span of the neuron in the image. Then Vaa3D-Neuron2 invokes an optimized all-path pruning algorithm^27^ to trace the neuron automatically (Methods and Supplementary Movie 12). When an image has a high SNR, for example, there is only one clearly labelled neuron in the image^28^,^29^, Vaa3D-Neuron2 may trace a neuron without any predefined landmark. When the SNR is very low, for example, some neurite regions might appear to be very dark or broken, Vaa3D-Neuron2 allows the use of PPA to define additional prior 3D landmarks to assist the tracing (Fig. 8d and Supplementary Fig. 3).

We have applied Vaa3D-Neuron2 to a wide variety of neuronal images. The software produces highly consistent reconstructions of a set of ten *Drosophila* projection neurons, despite independently defined initial spans for neuron reconstruction (Fig. 8a). Although each of these neurons has an uneven distribution of voxel intensity, small differences between the reconstructions typically occur only around the 3D landmarks manually defined by a user’s mouse clicks (Fig. 8a). For this data set, Vaa3D-Neuron2 is able to achieve sub-voxel tracing precision defined based on the repeatability of independent runs. This precision is ~13-fold better than the manual reconstruction achieved with the Neurolucida software (MBF Bioscience) (Fig. 8b and Supplementary Fig. 4). At this precision level, Vaa3D-Neuron2 reduces the amount of information that have been manually input by 5.4 times compared with Vaa3D-Neuron1 (ref. 8) (Supplementary Fig. 3). The running time of Vaa3D-Neuron2 to trace a neuron from this data set varies from 1.7 to 95 s, depending on the complexity of images (Supplementary Fig. 5). This is about 10~100 times faster than Vaa3D-Neuron1 and >1,000 times faster than pure manual reconstruction. Vaa3D-Neuron2 also successfully traces a data set of 22 dragonfly thoracic neurons that have very complicated arborization and substantial image noise resulting from the neuron labelling methodology (intracellular dye injection, Fig. 8c). For this data set, the computational cost of Vaa3D-Neuron2 is two to three orders of magnitude less than that of several alternative methods (Fig. 8d). Indeed, the new software package only needs 2~65 s to reconstruct a neuron (Supplementary Fig. 6), whereas manually reconstructing any one of these neurons takes a day or two.

In the proof-editing step, Vaa3D-Neuron2 uses CDA2 to correct imperfect parts in a traced neuron. We added a missing neuron segment or replaced an imperfect segment using a 3D curve produced via a single mouse stroke. When the starting or ending location of a newly added segment is close to existing neuron segments (at most 5-voxel apart), Vaa3D-Neuron2 automatically joins these segments. We also designed additional ways to refine a neuron segment, for example, dragging a curve in 3D. With this approach, an imperfect neuron structure is corrected quickly (Supplementary Movie 13).

**Figure 9:**
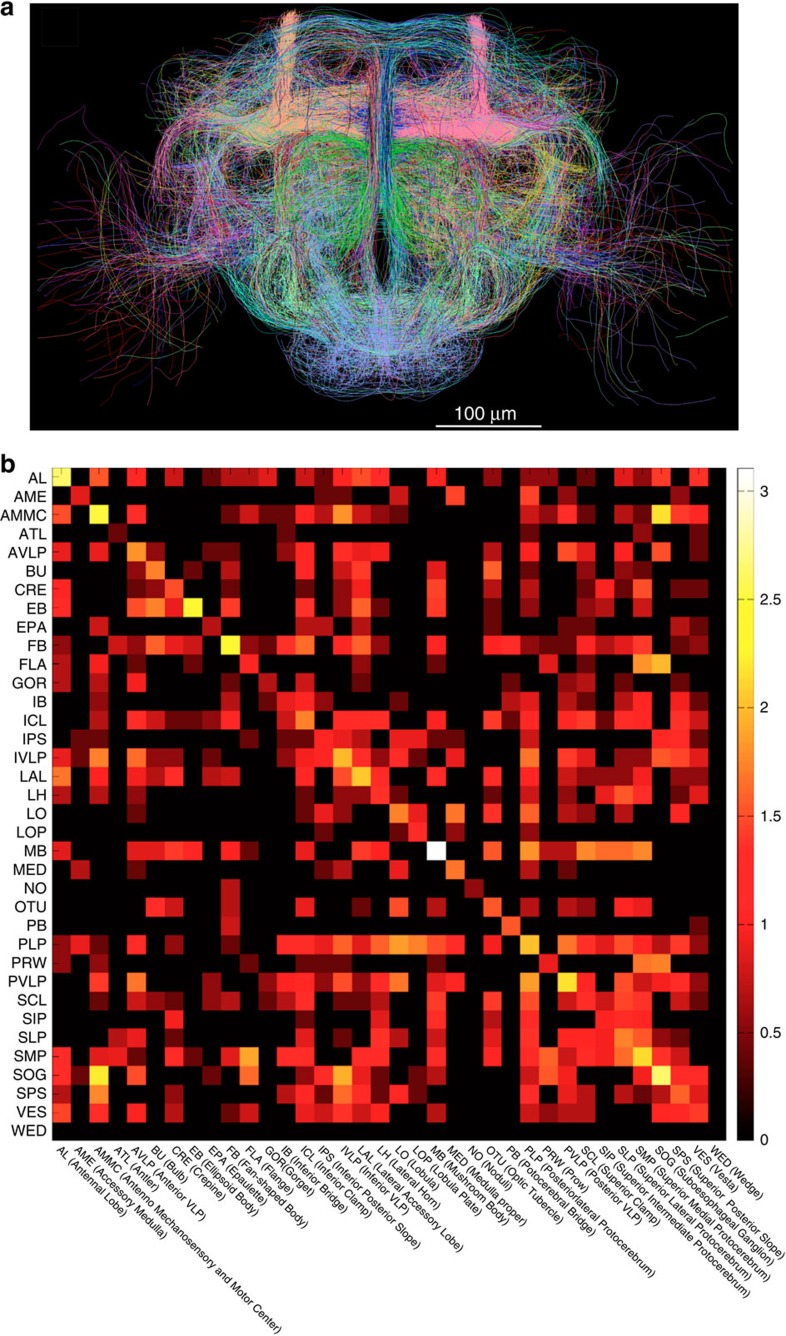
A whole *Drosophila*-brain projectome of neuron tracts. (**a**) Neuron tracts (9,198) extracted from 3D-registered confocal images of 1,107 GAL4 lines. The tracts that connect the same starting and ending brain compartments are colour matched. (**b**) The simplified projectome of neuronal patterns among different brain compartments. Scale bar, log_10_ of the number of projections between compartments.

The VF functions also allowed us to construct a projectome of the very complicated neuronal wiring of a *Drosophila* (fruit fly) brain within a few weeks. We screened more than 30,000 spatially aligned 3D confocal image stacks of ~7,000 transgenic GAL4 lines^13^,^30^. This effort identified 1,107 lines, each of which expresses a clear sub-population of neurons in the fruit fly brain. To analyse the labelling seen in the images of these GAL4 lines, we used Vaa3D-Neuron2 on a computer equipped with a touch screen and a digitizer pen, and traced 9,198 neurite fibre bundles that project among different brain compartments (Fig. 9a, Supplementary Movie 14 and Supplementary Data). This resulted in a detailed 3D digital projection map covering all known compartments of a *Drosophila* brain (Fig. 9a,b and Supplementary Fig. 7). This projectome exhibits an overall left–right symmetry (Fig. 9a). Several brain compartments display a high number of projections, including the mushroom bodies, antennal lobes, antennal mechanosensory and motor centre, and sub-oesophageal ganglion (Supplementary Fig. 7). These results indicate that these compartments may be the major centres of neuronal information processing in a *Drosophila* brain.

**Figure 10:**
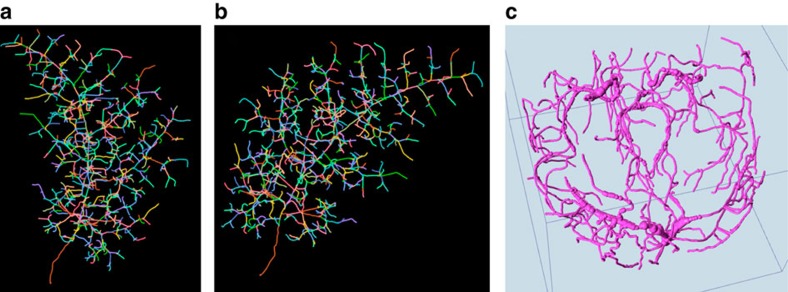
Vaa3D-Neuron2 reconstructions for other biological and biomedical applications. (**a**,**b**) The 3D reconstructed bronchial tree of a mouse lung from two view angles. (**c**) The 3D reconstruction of a human brain angiogram. See refs 40, 41 for exemplar details of the raw images and their biological applications for developmental biology, stem cell and human anatomy.

We also examined the applicability of VF and Vaa3D-Neuron2 to biology and biomedical fields other than neuroscience. We used Vaa3D-Neuron2 to reconstruct the complicated 3D bronchial tree (Fig. 10a,b) of a mouse lung filled using fluorescently labelled agarose. With Vaa3D-Neuron2, we needed to specify only 28 termini, followed by ~7 min of automated tracing on a Mac laptop computer, to produce a full reconstruction of the bronchial tree that was then validated by human. For this data set, Vaa3D-Neuron2 was ~17 times faster than Vaa3D-Neuron1, as this bronchial tree contains ~470 termini, all of which had to be manually specified in the Vaa3D-Neuron1 case. We also used Vaa3D-Neuron2 to reconstruct the major angiogram of a human brain (Fig. 10c) within 6 min. With conventional 2D tools, this task normally requires a few days of manual work to accomplish (cng.gmu.edu/brava).

## Discussion

In this study, we have proposed the VF method and demonstrated that it complements conventional approaches for exploring the content of an image. Our 3D VF technique enables real-time random access of 3D image space and captures key objects such as cells and tissue of interests that are useful in many biology studies. This technique was designed to use salient image features, such as 3D curves, and thus it works very robustly even for images with low SNRs. As a result, VF provides efficient and ergonomic ways to access bioimage content. The technique makes it easy to generate rich 3D prior information to guide image analysis and to proof edit the results of image computation. This technique also greatly facilitates adaptive microimaging and instant 3D free-form microsurgery. VF can also be applied to other important bioimaging applications. For instance, it can be used to specify any 3D area of interest when visualizing and managing a massive amount of voxel data. Vaa3D-TeraFly uses VF to achieve instant Google-Earth-like 3D visualization of terabytes, and virtually infinite amount, of high-resolution microscopic data for the first time. It also allows instant annotation of any visible 3D structures in the images. These features allow annotating whole-mouse brains or other similar *in toto* imaging data sets scanned at the sub-micron resolution.

The efficacy of VF for high-throughput, large-scale 3D image analysis is well illustrated by neuron tracing and proof editing in Vaa3D-Neuron2. The previous Vaa3D-Neuron1 system needed a user to pinpoint all termini of a neuron for tracing and was time-consuming for complicated neuron structures and noisy images. In contrast, Vaa3D-Neuron2 requires a minimal amount of prior information even for these challenging situations. 3D pinpointing and curve generation functions are also applicable to other image analysis tasks. For instance, in a 3D-imaging study of cell division^31^, the user could pinpoint 3D locations of kinetochores and trace the microtubules using CDA2, followed by effective tracking of such dynamically changing objects throughout a cell cycle. These applications show that VF is complementary to the automated image analysis. Their combination can allow more efficient and precise solutions for large-scale quantitative biology.

The application of VF to microimaging is not limited to confocal or multiphoton microscopy. It can be combined with other fast imaging methods such as SPIM^18^,^20^,^21^,^22^,^23^, which may be used for both pre-imaging and zoom-in imaging. As SPIM images the entire volume of a specimen rapidly, combination of VF and SPIM in one system could also be valuable for both the imaging of very large specimens and design of new functional imaging experiments. For large specimens, VF can help identify good ROIs in the pre-scanned image, and thus the zoom-in imaging can be selective to these spatially confined regions. In this way, the entire imaging system can be made even faster than using SPIM alone. With regards to the design of functional experiments, VF may be combined with the microsurgery system developed previously and based on SPIM^22^. Further, VF may be combined with other imaging systems such as Micropilot^6^, ScanImage^4^, μManager^5^ and Zen (Zeiss) to produce prior knowledge-driven automated imaging, which is useful for systems biology and a number of other biology domains. VF may also be useful for electron microscopy^32^, where dark regions in an image might correspond to signal. In this case, VF can be extended to minimum intensity projection or alpha-blending view of a 3D image.

The single-cell resolution 3D bleaching experiment for live *C. elegans* and 3D microsurgery of a fruit fly’s neuronal arbor offer interesting possibilities for various experiments in model animals. One potential use of VF is to combine the technique with optogenetics experiments^33^. The precise localization capability of VF makes it possible to introduce local perturbations of ion influx via channelrhodopsin and then quickly record the response (for example, through calcium imaging) of the live specimen under imaging. In combination with other genetic methods, it is possible to flexibly manipulate single cells or even smaller organelles. VF will also allow new alternatives for similar laser surgery and microdissection studies^34^,^35^, which are often limited to 2D specimens or easily accessible 3D locations. Similarly, VF can also be applied to enhance imaging or visually guided electrophysiology such as *in vivo* patch clamping^36^,^37^.

We showed that VF could be used to generate valuable data sets for neuroscience. For instance, our version of the projectome of a *Drosophila* brain complements a previous study based on a random mosaic of a large number of single neuron reconstructions (flycircuit.org)^38^. Importantly, as each of the reconstructed neurite tracts in our projectome is unambiguously associated with the specific fragments of some gene enhancers^39^, an analysis of this projectome might provide information about the abundance of gene expression in neuron populations and their wiring, which would otherwise be unavailable. Of note, although this article focuses on the technological aspects of VF instead of the new biological findings resulting from its various applications, it is nonetheless important to emphasize that the efficient reconstruction of 3D neuron morphology is a fundamental enabling technology for high-throughput quantitative neuroscience and can lead to a number of new biological insights regarding neuronal cell types, neuronal physiology, neuronal networks and associations between neurons’ intrinsic properties and animal behaviours^29^. In addition to neuroscience applications, VF and Vaa3D-Neuron2 can also find a wide range of uses in other fields that study fibrous, tree-like or networked bio-entities, such as the morphogenesis in developmental biology^40^ and anatomy of arterial vasculature^41^.

VF complements prevailing stereo 3D-display techniques and 3D human–machine interaction methods, such as 3D monitor and Kinect (Microsoft), respectively. VF inversely maps the selection of a locus on the 2D projection plane to the 3D volumetric space of an image stack. Stereo 3D display generates the stereo view of a 3D scene. The Kinect-like technique is essentially a 3D motion sensor. It is possible to combine these three techniques in one future system to allow a user to see stereo-displayed 2D projections of a 3D image, and use 3D motion instead of drawing on 2D planes to add more cues of 3D prior information generated by a human. Such a future system may offer users a more realistic interaction experience of 3D image content.

VF works robustly and precisely in general to estimate the most probable solution of the inverse problem to find 3D locations of objects in images based on a minimal amount of input in the 2D projection planes. Yet, VF could fail if an object is invisible in a 3D rendered view such as some alpha-blended displays, or the object is made visible only after applying certain filtering. For the former situation, the solution should be to use a sub-volume maximal intensity projection rendering to make the object visible, followed by VF. For the latter situation, the solution is to apply the same filtering to tuning of the VF algorithms, thus to make the human perception and the machine-computed results be consistent. VF has been designed most for fluorescently labelled data or similar images (for example, bright-field images). The extension of this method for other imaging modalities such as electron microscopy data can be an interesting future topic.

Finally, VF may enhance many existing bioimaging software packages^4^,^5^,^6^,^7^,^8^,^9^,^10^,^11^,^12^, which use various automated and semi-automated methods to acquire, visualize, interact, analyse and manage bioimage data. VF can be a valuable intersection and enhancement of all these very different topics that are normally studied under dissimilar scenarios. As a proof of principle, we have incorporated this technique as a module in our latest Open Source Vaa3D system (vaa3d.org). This module and its various algorithms are reusable for other bioimaging-related tools. The VF techniques are starting to be used in several large-scale bioimage informatics-related initiatives such as the Allen Institute’s MindScope project and the *European Human Brain Project*.

## Methods

### Curve drawing and refining

A curve is defined by an ordered series of knots, each of which has a 3D location. 2D input devices, for example, a computer mouse or a digitizer pen, have a limited sampling rate on the 2D screen, as well as some inaccuracy due to shaking of a user’s hand or other factors. Thus, to generate a 3D curve robustly, we mapped each sampled point in an input device’s on-screen trajectory to the 3D volumetric space of an image, and at the same time cope with the inaccuracy of such 2D sampling points.

We generated a 3D curve in a piece-wise manner. Each piece is defined as a short 3D curve segment that connects a 3D starting point (*p*_*k*_ in Figs 1 and 2), which corresponds to the 3D location of the last estimated curve knot, to a 3D ending point (*p*_*k*+1_ in Fig. 1), which should be estimated based on the current shooting-ray *R*_*k*+1_ (Fig. 1).

To do so, CDA2 finds the shortest geodesic path from *p*_*k*_ to all voxels on the ray *R*_*k*+1_. The ‘geodesic’ distance between two immediately adjacent voxels *v* and *u* is defined as


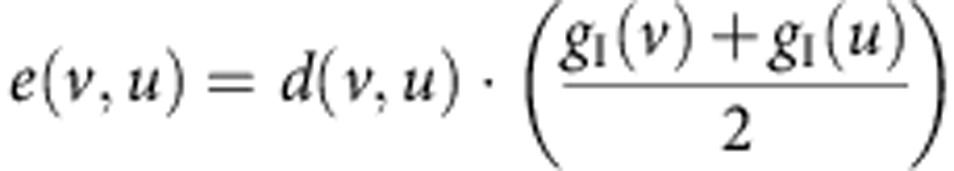


where the first item in the product is the Euclidean distance of the two voxels, and *g*_I_(.) in the second item has the following form,


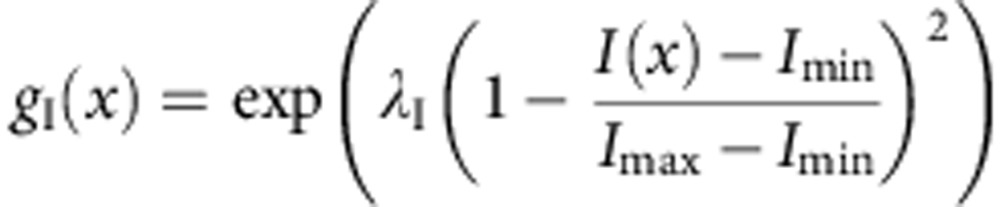


where λ_I_ is a weighting factor (set as 10 by default), *I*(*x*) is intensity of the voxel *x*, *I*_min_ and *I*_max_ are the minimal and maximal intensity in the image, respectively.

With this geodesic distance function, we have actually treated an image as a graph: each voxel is coded as a graph node; only spatially adjacent (see below) voxel nodes have edges; the weight of each edge is defined using the above geodesic distance score.

The shortest path between a node and the remaining nodes in this graph could be produced by invoking the Dijkstra algorithm such as shown in the ref. 8. However, for the purposes to (a) avoid the overhead of generating an explicit graph data structure that may have a very large size for a big image and also (b) gain more flexibility in finding the shortest path between any two sets of graph nodes, here we adapted a fast-marching algorithm^42^ that propagates from a set of source voxels to a set of sink voxels. This fast-marching scheme is essentially a 3D region-growing method equivalent to the breadth-first graph search algorithm. In our software, to determine the ‘adjacent’ voxels, a user may choose using the 6-connected, 18-connected or 26-connected neighbours, where we set the default to be 18-connected neighbours, and have noted that practically the three choices yielded similar results.

In the simplest case of CDA2, the source-voxel set contains only *p*_*k*_, while the sink-voxel set contains all voxels immediately around the ray *R*_*k*+1_. The first hit-point on *R*_*k*+1_ is chosen as *p*_*k*+1._ For the initial knot on the first shooting ray, the fast-marching algorithm is applied between all points between the first and second shooting rays to find out the shortest path and thus the two termini at the same time. Finally, the entire curve is smoothed using a small moving window (about 5-voxel wide in most cases). Notably, at each step the 3D location of a curve knot has been optimally corrected to avoid accumulated error.

We have added the new curve drawing functions on top of the previously published Open Source Vaa3D system (vaa3d.org)^8^. In the implementation, we also added a few more components to make CDA2 more robust, more precise, and faster. First, we noticed that a computer mouse often has an uneven sampling rate when it moves on screen, and sometimes the hand of a human operator may have sudden shaking. Thus, we preprocessed the trajectory of a mouse stroke to use only 50% of its on-screen sampling points that correspond to the brightest voxels.

Second, we considered eliminating the potential problems such as depending on an inaccurate starting curve-knot *p*_0_ or noisy bright voxels in intermediate rays. To do that, for each pair of consecutive sampling points on the screen, we generated two rays and then used the fast marching method to produce the shortest path between them. Next, we searched for the globally shortest path that ran through all consecutive rays, allowing us to choose the global minimum cost path starting from the first ray and ending at the last ray, in all possible combinations of initial paths through consecutive rays (Fig. 2 and Supplementary Movie 5).

As a variant of the above CDA2 method, we also considered restricting the search area of the shortest paths of a pair of consecutive rays to the 3D bounding box defined by the two 3D points first determined using PPA (Fig. 2). This method, however, often generates comparable results with the above CDA2 implementation.

We also designed several other methods to refine an existing 3D curve. One method allows a user to drag some knots of a 3D curve and smoothly deform the curve. Another method allows for refining some region of a curve using more mouse strokes. The third method allows for shifting, rotating, mirroring and zooming a 3D curve directly. This third method is often very useful especially when a user wants to compare or edit collections of curves (for example, neuron morphology) that have different scalings, orientations and displacements.

### 3D pinpointing for multiple colour-channel data and noisy data

For a multicolour image (Fig. 3), we first estimated the best candidate 3D locations independently for each data channel in the image. Next, we picked from all candidates the location with the brightest voxel intensity as the most probable point that the user is pinpointing. Our software also allows a user to specify a particular colour channel for 3D pinpointing, instead of using all colour channels.

For a highly noisy image, we generated a robust estimate of the 3D pinpointing location based on 3D curve drawing. When a user presses the computer mouse, our system detects the very short path of mouse movement. Next, the 3D curve drawing algorithm is applied to detect the 3D location of this short path. The starting point of such a path is returned as the 3D pinpointing location.

### Ground–truth curve and reconstruction

The ‘ground–truth’ curves were generated by first using the Vaa3D-Neuron1 system^8^, which lets a user specify the two end points of a curve and then generates a smooth 3D curve. The user can then overlay the curve on top of the image in 3D as a proofreading measure to ensure its correctness. The SD score^8^ was computed using the Vaa3D package.

### Imaging system

The prototype of the SmartScope system is a home-built laser scanning confocal microscope, based on an upright microscope frame (BX51WI, Olympus). Both 488 nm (Spectra-Physics Cyan, 50 mW, Newport Corp.) and 561 nm (25 mW, CrystaLaser) lasers are used as excitation light sources. Another independently controlled 488 nm laser (Spectra-Physics Cyan, 100 mW, Newport Corp.) is used for bleaching and microsurgery purposes, and its beam is incorporated into the excitation light path by a beamsplitter. A non-resonant 2D scanner (Cambridge Technology) is used to locate laser foci at any position in the image plane, while a Piezo-based objective focusing system (P-721.CDQ, Physik Instrumente) enables axial variation of focal plane inside the sample. A 40 × 1.15 numerical aperture water-immersion objective (UApo/340, Olympus) is used to focus the laser beams and collect fluorescent signal. In the fluorescence detection light path, the fluorescence is focused and passes through a pinhole, and is recollimated and seperated into two channels (green and red) via a dichroic filter (567LP, Thorlabs) and bandpass filters (520/35, 617/73, Semrock), respectively. Two PMT detectors (H7422A-40, Hamamatsu) are used to convert the fluorescence intensity detected in both channels into electronic signal. The imaging system is controlled through multifunctional data acquisition boards (PCI6115, PCI6024E, USB-6229, National Instruments) and by a Vaa3D plugin, SmartScope-controller, which was developed in C/C++ language and linked with NIDAQ library (National Instruments). The SmartScope controller is able to randomly access any series of 3D points that fall into the field of view of the objective. The imaging system is called ‘SmartScope’ as the ROI for experiments can be directly determined either automatically or semi-automatically by 3D image analysis modules. The SmartScope controller programme is available for collaborative research.

### Specimen preparation

For fixed specimens, we used the L1 stage *C. elegans* with Punc-54::H1::cherry and Pmyo3::eGFP following the protocol in ref. 11, and the adult *Drosophila* brains with ato-GAL4 following the protocol in ref. 13. For live samples, we used L1 stage *C. elegans* treated with 0.1 mM levamisole in M9 buffer. This reversibly immobilizes the animals for up to 1 h.

### Massive volumetric image data visualization and annotation

Vaa3D-TeraFly was implemented as a Vaa3D plugin programme. It uses VF and a multi-resolution/multi-scale octree representation of an image stack (similar to the image representation in HDF5) to explore and annotate massive volumetric image data smoothly. To generate the octree, we designed a separate computer programme called TeraConverter, which starts with a full-resolution image stack (or equivalently a series of individual 2D image sections). TeraConverter iteratively downsamples twice for each of the X, Y and Z dimensions until a preset small volume (typically, 512 × 512 × 256 voxels) has been reached. The final (coarsest level) and intermediate smaller volumes are all saved to files and indexed using a tree data structure. In the tree, each node stands for an image block. The root node corresponds to the coarsest level and has eight child nodes. Similarly, any of the branching nodes in the octree corresponds to an image block with intermediate resolution and has eight child nodes. After the octree representation has been generated, Vaa3D-TeraFly is able to navigate the big 3D volume quickly: when the user zooms in with the mouse click, mouse stroke or mouse wheel into an image at the displayed resolution, an ROI is generated using VF and the respective image content is loaded from the higher-resolution image files quickly. To generate the ROI in the first two cases, PPA_*c*_ and CDA1 are invoked, respectively. In the third case, the ROI is generated based on using multiple rays sampled from the current view port that displays the 3D image. For each ray, the PPA_*c*_ method is applied to automatically generate a 3D location. Finally, the bounding box of all such generated locations is used as the ROI for the zoom-in operation. At all resolutions, image data are stored according to a tile-wise organization linked by the octree. Vaa3D-TeraFly then uses the Vaa3D’s 3D-rendering features to display the loaded data. Moreover, it conveniently integrates the Vaa3D’s 3D annotation features, which is also based on VF, at the different scales.

The machines used for bench testing the Vaa3D-TeraFly are a MacBook Pro Retina laptop with 2.7 GHz Intel Core i7, 16 GB 1,600 MHz DDR3 memory and NVIDIA GeForce GT 650 M 1,024 MB graphics card running Mac OS X 10.9, a Linux CentOS desktop with Intel Xeon 3.6 GHz CPU and Nvidia Quadro 2,000 graphics card, and a Dell desktop with Intel Xeon 3.33 GHz CPU and Nvidia GeForce GTX 480 graphics card running Windows 7 64 bit Professional Service Pack 1. All five test image stacks were hosted on a Seagate 4Tb external hard drive running USB 3.0 interface. The statistics for each test case were produced by on at least 20 trials of randomly selected target ROIs in arbitrarily determined scales of the respective image stack.

### Neuron reconstruction

Vaa3D-Neuron2 tracing can be done using zero, one, two or more landmark points, each of which is generated using one mouse operation. First of all, when there is only a single neuron in the image, Vaa3D-Neuron2 may detect the cell body and start the tracing process automatically, without any manual input (for example, mouse clicks). In a more complicated situation, such as when two or more neurons are present in one image or the single neuron is heavily contaminated by noise in an image, additional priors might be used. When one landmark point is used, Vaa3D-Neuron2 uses PPA to find the 3D landmark point corresponding to a mouse click, and then takes such a point as the seed to run the optimized all-path pruning algorithm^27^ to trace a neuron. When two or more landmarks are used, the first one will be used as the seed point for the automated tracing, which will finish when the second-to-last 3D landmark points have been reached.

In the proof-editing step, CDA2 is used to add a missing neuron segment or replace an imperfect neuron segment. In addition, over-traced neuron segments can be easily deleted using Vaa3D^8^.

## Author contributions

H.P. and F.L. conceived the project. J.T., J.Z. and H.P. built the SmartScope hardware and software. H.P. designed and conducted the imaging experiments with help from V.B. V.B. also prepared the *C. elegans* samples. H.P. and H.X. designed and implemented the 3D curve drawing algorithms and Vaa3D-Neuron2. J.Z. helped importing part of the curve drawing algorithms into Vaa3D. A.B., G.I. and H.P. designed and implemented the terabyte data visualization and annotation. Z.Z., P.T.G., S.W.O., J.C., A.M., R.W.T., H.Z. and G.A. provided various data sets and participated in discussion. E.M. and M.H. advised on this project. H.P. wrote the paper with input from all co-authors.

## Additional information

**How to cite this article:** Peng, H. *et al*. Virtual finger boosts three-dimensional imaging and microsurgery, and terabyte volume image visualization and analysis. *Nat. Commun.* 5:4342 doi: 10.1038/ncomms5342 (2014).

## Supplementary Material

Supplementary Figures Supplementary Figures 1-7

Supplementary Data 1 3D projectome and reconstructed neurite tracts of a *Drosophila* brain.

Supplementary Movie 1 3D point-pinpointing for a multicolor image. Image: *Drosophila* late embryo; cells are labeled using fluorophores with different colors.

Supplementary Movie 2 Robust 3D point-pinpointing based on a very short curve-drawing.

Supplementary Movie 3 Robust 3D curve-drawing for a noisy dragonfly confocal image.

Supplementary Movie 4 Stability test of CDA2 algorithm for different starting and ending locations, different brightness and different continuity levels of tract-like signal. In this video, different colors were used to indicate different curves that are generated for different starting and ending locations. We also used two tracts, one bright and one dark (also broken), for testing. It can be noticed that within the several produced curves for each tract, these curves correspond very well to each other at the locations where there is signal of a tract, despite the different starting and ending locations of the 2D drawing and the brightness and continuity of the tracts.

Supplementary Movie 5 Step-by-step illustration of the globally optimal CDA2 algorithm.

Supplementary Movie 6 An example of drawing a correct 3D curve using CDA2 for a relatively dark and partially broken tract obscured by another much brighter tract in the front.

Supplementary Movie 7 An example of drawing a correct 3D curve using CDA2 for a relatively dark and partially broken tract, which is obscured by another much brighter tract in the back.

Supplementary Movie 8 3D region-of-interest (ROI) generation using one mouse click or stroke. The identified ROI is displayed using a separate zoom-in 3D window.

Supplementary Movie 9 3D navigation of a 96Gb 3D image volume of rat hippocampal neurons based on Vaa3D-TeraFly's single computer-mouse zoom method, from the whole-neuron scale down to the single spine scale. For each zoom-in and zoom-out operation, Vaa3D-TeraFly utilizes the VF method to automatically detect the most probable 3D ROI the user is looking at and thereafter load and display the respective image data. Note that the method is robust for both bright and dark image regions. The test was done using a Mac laptop and an external hard drive.

Supplementary Movie 10 3D navigation of a 96Gb 3D image volume of rat hippocampal neurons based on Vaa3D-TeraFly's single computer-mouse stroke method. For each mouse-stroke (circling in this case) operation, Vaa3D-TeraFly utilizes the VF method to automatically detect the most probable 3D ROI the user is looking at and thereafter load and visualize the respective image data. The ROI-computing (based on single computer-mouse strokes) and respective 3D data rendering time are intentionally shown for a series of randomly chosen ROIs. Note that the method is robust for both bright and dark image regions. The test was done using a Mac laptop and an external hard drive.

Supplementary Movie 11 Labeling and annotation of neuron segments in a 96Gb 3D image volume of rat hippocampal neurons based on Vaa3D-TeraFly. The two exemplar neuron-segments can be identified and reconstructed correctly and easily in 3D, even the operations to define them happen in 2D. Such annotation is carried over across all different scales when the image data is loaded or refreshed. This makes it is very efficient to reconstruct, annotate, and proof-edit very large 3D image datasets. The test was done using a Mac laptop and an external hard drive.

Supplementary Movie 12 Very efficient neuron tracing using two landmark points, which define a rough initial span of the neuron for automated reconstruction.

Supplementary Movie 13 Efficient editing of a neuron's 3D morphology after 3D proof-reading.

Supplementary Movie 14 3D visualization of the *Drosophila* brain projectome. See Fig. 7a for more information about the data.

## References

[b1] Walter T. . Visualization of image data from cells to organisms. Nat. Methods 7, S26–S41 (2010). 20195255 10.1038/nmeth.1431 PMC3650473

[b2] Eliceiri K. W. . Biological imaging software tools. Nat. Methods 9, 697–710 (2012). 22743775 10.1038/nmeth.2084 PMC3659807

[b3] Long F., Zhou J. & Peng H. Visualization and analysis of 3D microscopic images. PLoS Comput. Biol. 8, e1002519 (2012). 22719236 10.1371/journal.pcbi.1002519 PMC3375219

[b4] Pologruto T. A., Sabatini B. L. & Svoboda K. ScanImage: flexible software for operating laser scanning microscopes. Biomed. Eng. Online 2, 13 (2003). 12801419 10.1186/1475-925X-2-13 PMC161784

[b5] Edelstein A., Amodaj N., Hoover K., Vale R. & Stuurman N. Computer control of microscopes using μManager. Curr. Protoc. Mol. Biol Chapter14, Unit14.20 (2010). 20890901 10.1002/0471142727.mb1420s92 PMC3065365

[b6] Conrad C. . Micropilot: automation of fluorescence microscopy-based imaging for systems biology. Nat. Methods 8, 246–249 (2011). 21258339 10.1038/nmeth.1558 PMC3086017

[b7] Abràmoff M. D., Magalhães P. J. & Ram S. J. Image processing with ImageJ. Biophoton. Int. 11, 36–42 (2004).

[b8] Peng H., Ruan Z., Long F., Simpson J. H. & Myers E. W. V3D enables real-time 3D visualization and quantitative analysis of large-scale biological image data sets. Nat. Biotechnol. 28, 348–353 (2010). 20231818 10.1038/nbt.1612 PMC2857929

[b9] Sommer C., Straehle C., Kothe U. & Hamprecht F. A. inBiomedical Imaging: From Nano to Macro. IEEE International Symposium on 230-233 IEEE (2011).

[b10] Carpenter A. E. . CellProfiler: image analysis software for identifying and quantifying cell phenotypes. Genome Biol. 7, R100 (2006). 17076895 10.1186/gb-2006-7-10-r100 PMC1794559

[b11] Long F., Peng H., Liu X., Kim S. K. & Myers E. A 3D digital atlas of *C. elegans* and its application to single-cell analyses. Nat. Methods 6, 667–672 (2009). 19684595 10.1038/nmeth.1366 PMC2882208

[b12] Lau C. . Exploration and visualization of gene expression with neuroanatomy in the adult mouse brain. BMC Bioinformatics 9, 153 (2008). 18366675 10.1186/1471-2105-9-153 PMC2375125

[b13] Peng H. . BrainAligner: 3D registration atlases of *Drosophila* brains. Nat. Methods 8, 493–498 (2011). 21532582 10.1038/nmeth.1602 PMC3104101

[b14] Peng H., Long F., Zhao T. & Myers E. Proof-editing is the bottleneck of 3D neuron reconstruction: the problem and solutions. Neuroinformatics 9, 103–105 (2011). 21170608 10.1007/s12021-010-9090-x

[b15] Betzig E. . Imaging intracellular fluorescent proteins at nanometer resolution. Science 313, 1642–1645 (2006). 16902090 10.1126/science.1127344

[b16] Rust M. J., Bates M. & Zhuang X. Sub-diffraction-limit imaging by stochastic optical reconstruction microscopy (STORM). Nat. Methods 3, 793–796 (2006). 16896339 10.1038/nmeth929 PMC2700296

[b17] Planchon T. A. . Rapid three-dimensional isotropic imaging of living cells using Bessel beam plane illumination. Nat. Methods 8, 417–423 (2011). 21378978 10.1038/nmeth.1586 PMC3626440

[b18] Tomer R., Khairy K. & Keller P. J. Light sheet microscopy in cell biology. Methods Mol. Biol. 123–137 (2013). 23027001 10.1007/978-1-62703-056-4_7

[b19] Liu X. . Analysis of cell fate from single-cell gene expression profiles in *C. elegans*. Cell 139, 623–633 (2009). 19879847 10.1016/j.cell.2009.08.044 PMC4709123

[b20] Huisken J., Swoger J., Del Bene F., Wittbrodt J. & Stelzer E. H. Optical sectioning deep inside live embryos by selective plane illumination microscopy. Science 305, 1007–1009 (2004). 15310904 10.1126/science.1100035

[b21] Dodt H.-U. . Ultramicroscopy: three-dimensional visualization of neuronal networks in the whole mouse brain. Nat. Methods 4, 331–336 (2007). 17384643 10.1038/nmeth1036

[b22] Engelbrecht C. J. . Three-dimensional laser microsurgery in light-sheet based microscopy (SPIM). Opt. Express 15, 6420–6430 (2007). 19546948 10.1364/oe.15.006420

[b23] Preibisch S., Saalfeld S., Schindelin J. & Tomancak P. Software for bead-based registration of selective plane illumination microscopy data. Nat. Methods 7, 418–419 (2010). 20508634 10.1038/nmeth0610-418

[b24] Ragan T. . Serial two-photon tomography for automated ex vivo mouse brain imaging. Nat. Methods 9, 255–258 (2012). 22245809 10.1038/nmeth.1854 PMC3297424

[b25] Donohue D. E. & Ascoli G. A. Automated reconstruction of neuronal morphology: an overview. Brain Res. Rev. 67, 94–102 (2011). 21118703 10.1016/j.brainresrev.2010.11.003 PMC3086984

[b26] Helmstaedter M. & Mitra P. P. Computational methods and challenges for large-scale circuit mapping. Curr. Opin. Neurobiol. 22, 162–169 (2012). 22221862 10.1016/j.conb.2011.11.010 PMC3406305

[b27] Peng H., Long F. & Myers G. Automatic 3D neuron tracing using all-path pruning. Bioinformatics 27, i239–i247 (2011). 21685076 10.1093/bioinformatics/btr237 PMC3117353

[b28] Yu H. -H., Chen C. -H., Shi L., Huang Y. & Lee T. Twin-spot MARCM to reveal the developmental origin and identity of neurons. Nat. Neurosci. 12, 947–953 (2009). 19525942 10.1038/nn.2345 PMC2701974

[b29] Gonzalez-Bellido P. T., Peng H., Yang J., Georgopoulos A. P. & Olberg R. M. Eight pairs of descending visual neurons in the dragonfly give wing motor centers accurate population vector of prey direction. Proc. Natl Acad. Sci. 110, 696–701 (2013). 23213224 10.1073/pnas.1210489109 PMC3545807

[b30] Jenett A. . A GAL4-driver line resource for Drosophila neurobiology. Cell Rep. 2, 991–1001 (2012). 23063364 10.1016/j.celrep.2012.09.011 PMC3515021

[b31] Thompson S. L. & Compton D. A. Chromosome missegregation in human cells arises through specific types of kinetochore–microtubule attachment errors. Proc. Natl Acad. Sci. USA 108, 17974–17978 (2011). 21997207 10.1073/pnas.1109720108 PMC3207692

[b32] Bozzola J. J. & Russell L. D. Electron Microscopy: Principles and Techniques for Biologists Jones & Bartlett Learning (1999).

[b33] Deisseroth K. Optogenetics. Nat. Methods 8, 26–29 (2010). 21191368 10.1038/nmeth.f.324 PMC6814250

[b34] Berns M. W. . Laser microsurgery in cell and developmental biology. Science 213, 505–513 (1981). 7017933 10.1126/science.7017933

[b35] Fang-Yen C., Gabel C. V., Samuel A. D., Bargmann C. I. & Avery L. Laser microsurgery in Caenorhabditis elegans. Methods Cell Biol. 107, 177 (2012). 22226524 10.1016/B978-0-12-394620-1.00006-0 PMC3617498

[b36] Wang X. & Li M. Automated electrophysiology: high throughput of art. Assay Drug Dev. Technol. 1, 695–708 (2003). 15090242 10.1089/154065803770381057

[b37] Kodandaramaiah S. B., Franzesi G. T., Chow B. Y., Boyden E. S. & Forest C. R. Automated whole-cell patch-clamp electrophysiology of neurons *in vivo*. Nat. Methods 9, 585–587 (2012). 22561988 10.1038/nmeth.1993 PMC3427788

[b38] Chiang A.-S. . Three-dimensional reconstruction of brain-wide wiring networks in drosophila at single-cell resolution. Curr. Biol. 21, 1–11 (2011). 21129968 10.1016/j.cub.2010.11.056

[b39] Pfeiffer B. D. . Tools for neuroanatomy and neurogenetics in *Drosophila*. Proc. Natl Acad. Sci. 105, 9715–9720 (2008). 18621688 10.1073/pnas.0803697105 PMC2447866

[b40] Metzger R. J., Klein O. D., Martin G. R. & Krasnow M. A. The branching programme of mouse lung development. Nature 453, 745–750 (2008). 18463632 10.1038/nature07005 PMC2892995

[b41] Wright S. N. . Digital reconstruction and morphometric analysis of human brain arterial vasculature from magnetic resonance angiography. NeuroImage 82, 170–181 (2013). 23727319 10.1016/j.neuroimage.2013.05.089 PMC3971907

[b42] Sethian J. A. Level set methods and fast marching methods: evolving interfaces in computational geometry, fluid mechanics, computer vision, and materials science. in: *Cambridge Monographs on Applied and Computational Mathematics* Vol. 3, (Cambridge University Press (1999).

