# Supplementary material for: Virtual finger boosts three-dimensional imaging and microsurgery as well as terabyte volume image visualization and analysis
Source: Nat Commun. 2014 Jul 11;5:4342. doi: 10.1038/ncomms5342 (PMC4104457; doi:10.1038/ncomms5342)
Supplement: Supplementary Figures — 1-7 [file ncomms5342-s1.pdf]

Supplementary Figures

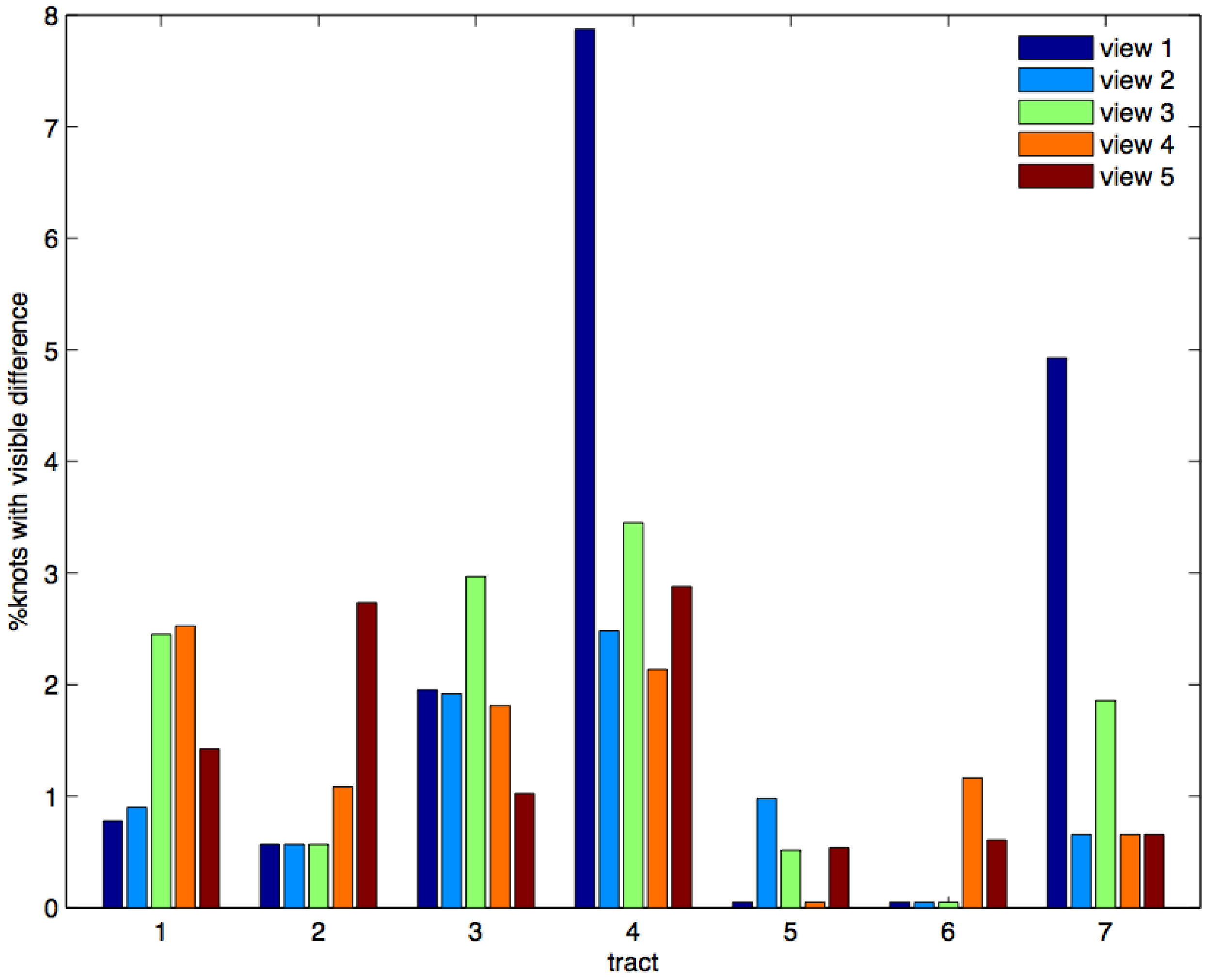

(a)

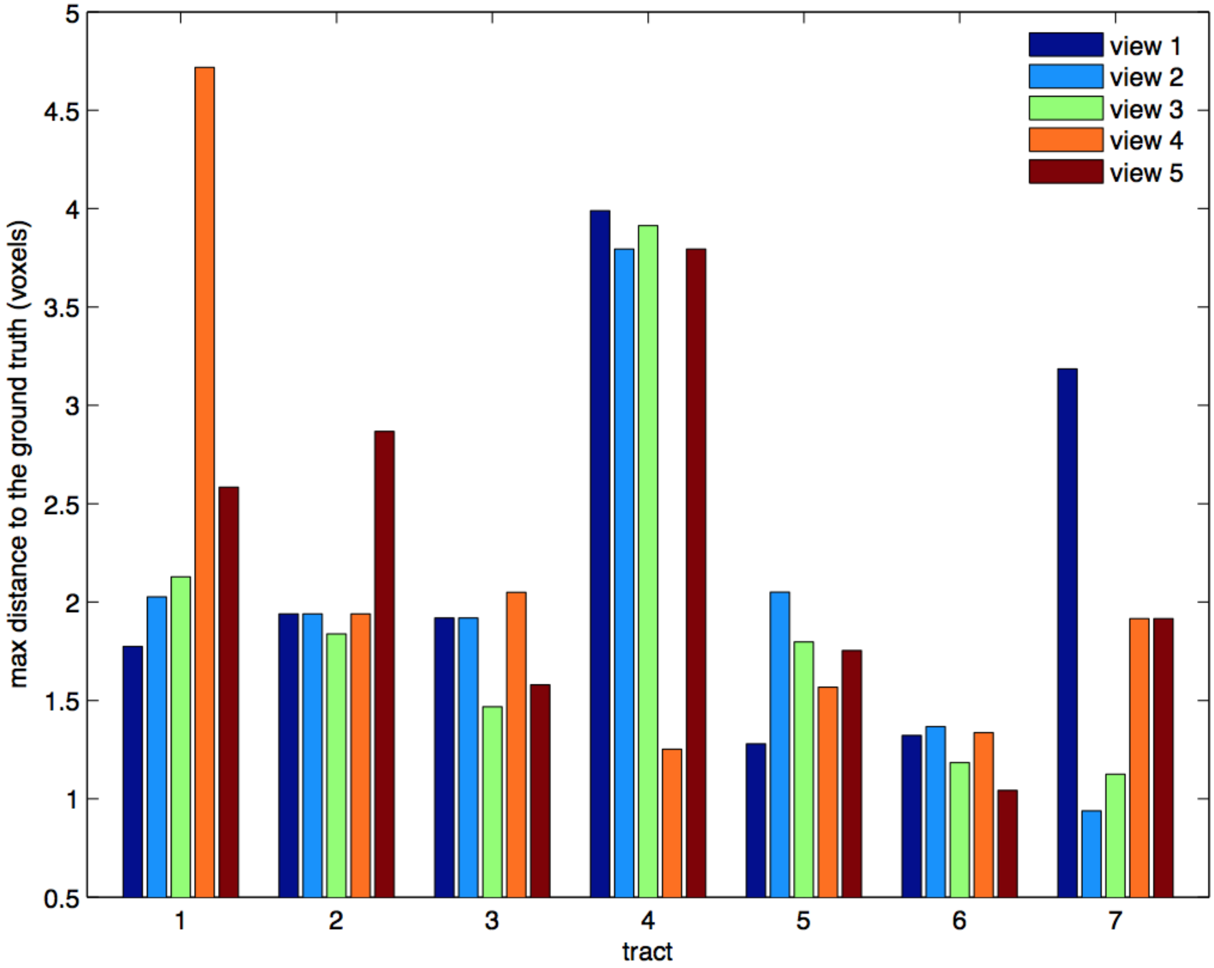

(b)

Supplementary Fig. 1. (a) Precision of CDA curve drawing indicated by the percentage of curve-knots that have visible spatial difference (≥ 2 voxels) when each of the tracts (curves) is compared with its corresponding ground truth. (b) The maximal spatial distance between individual tracts and the respective ground truth curves. Note that while the maximal values in (b) provide upper bounds for the spatial divergence of the tracts, the total percentages of curve-knots that actually have visible spatial difference are very small (as indicated in (a)). For both (a) and (b), the data is based on 1,470 measurements of 7 tracts.

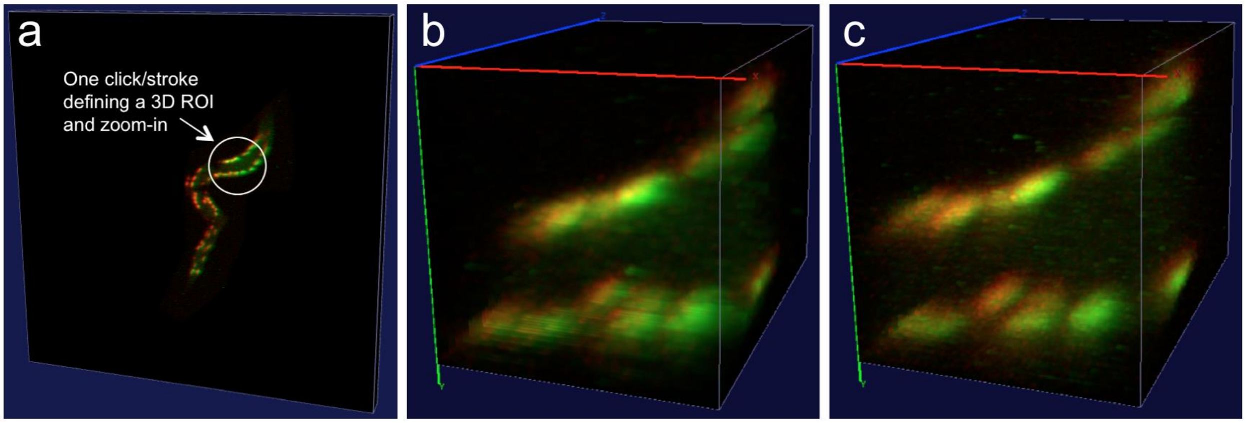

Supplementary Fig. 2. Instant 3D zoom-in imaging of single-nucleus gene expression for *C. elegans*. (a) A 3D pre-scan image of L1 *C. elegans*. Circle: an ROI defined by one mouse stroke. (b) Digital zoom-in of the ROI, where the insufficient voxel resolution does not manifest clear nuclei boundary. (c) Optical zoom-in around the ROI, where the boundary between several nuclei is visible.

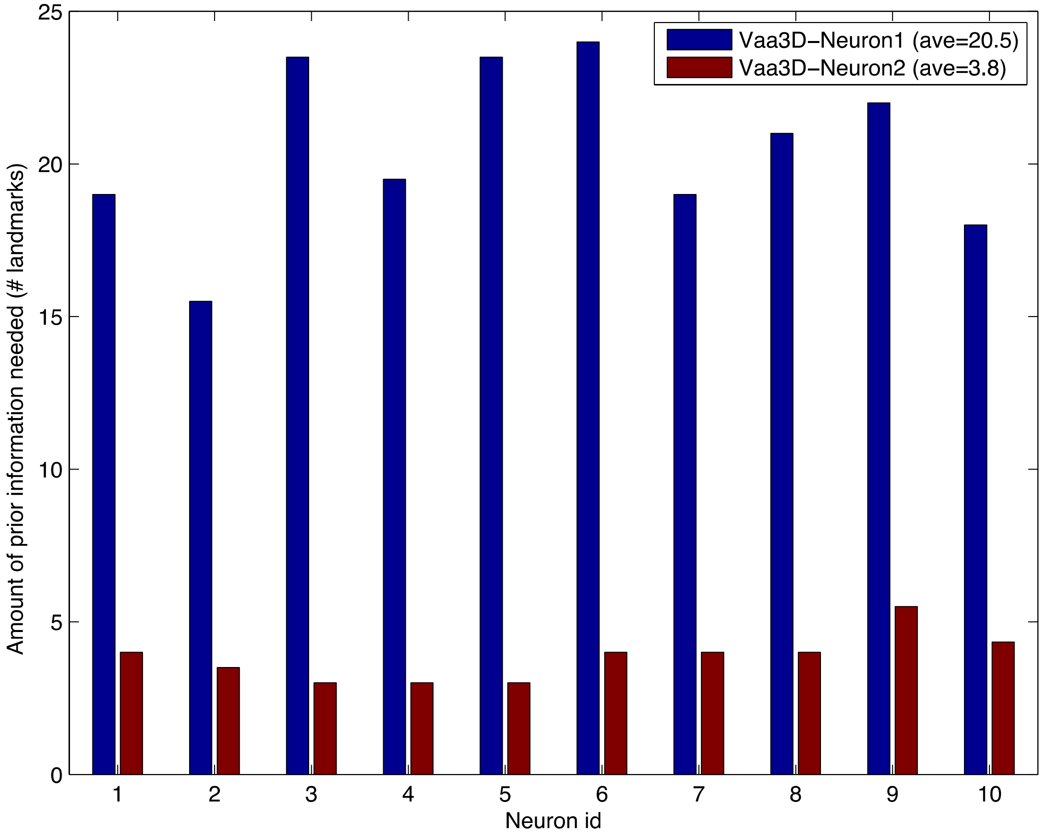

Supplementary Fig. 3. Amount of prior information needed for reconstructing 10 *Drosophila* neurons.

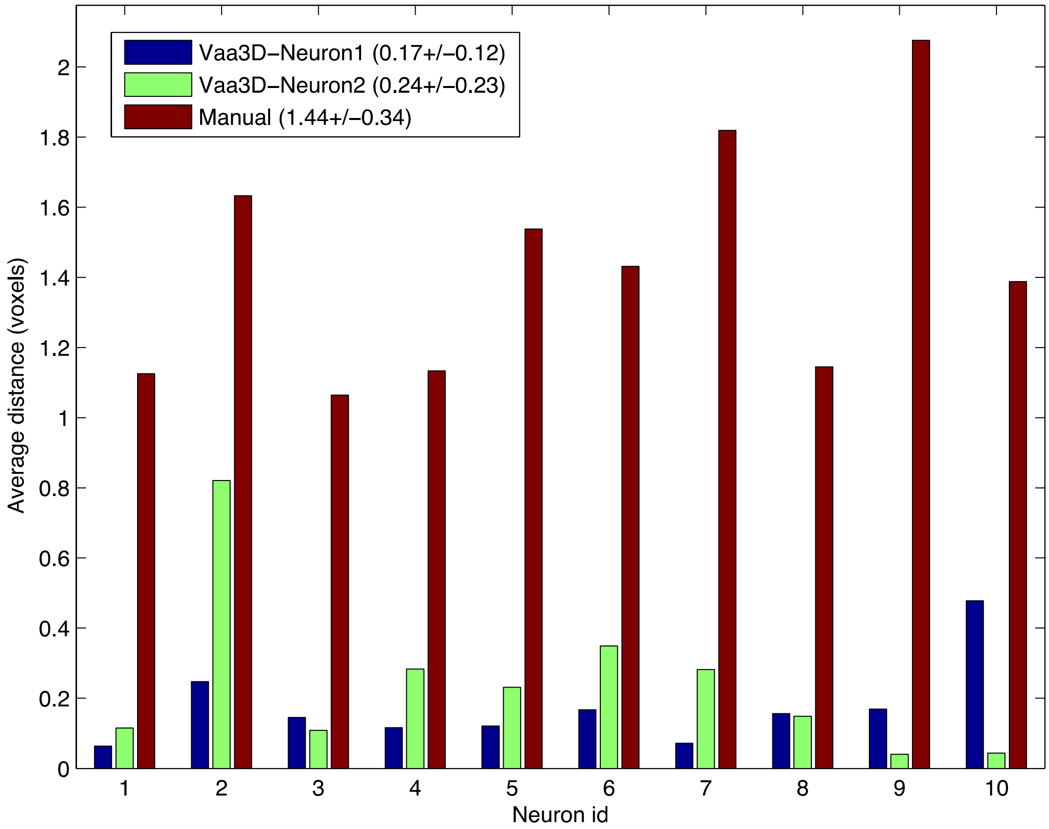

Supplementary Fig. 4. Precision of reconstruction of 10 *Drosophila* projection neurons; each neuron was reconstructed twice independently.

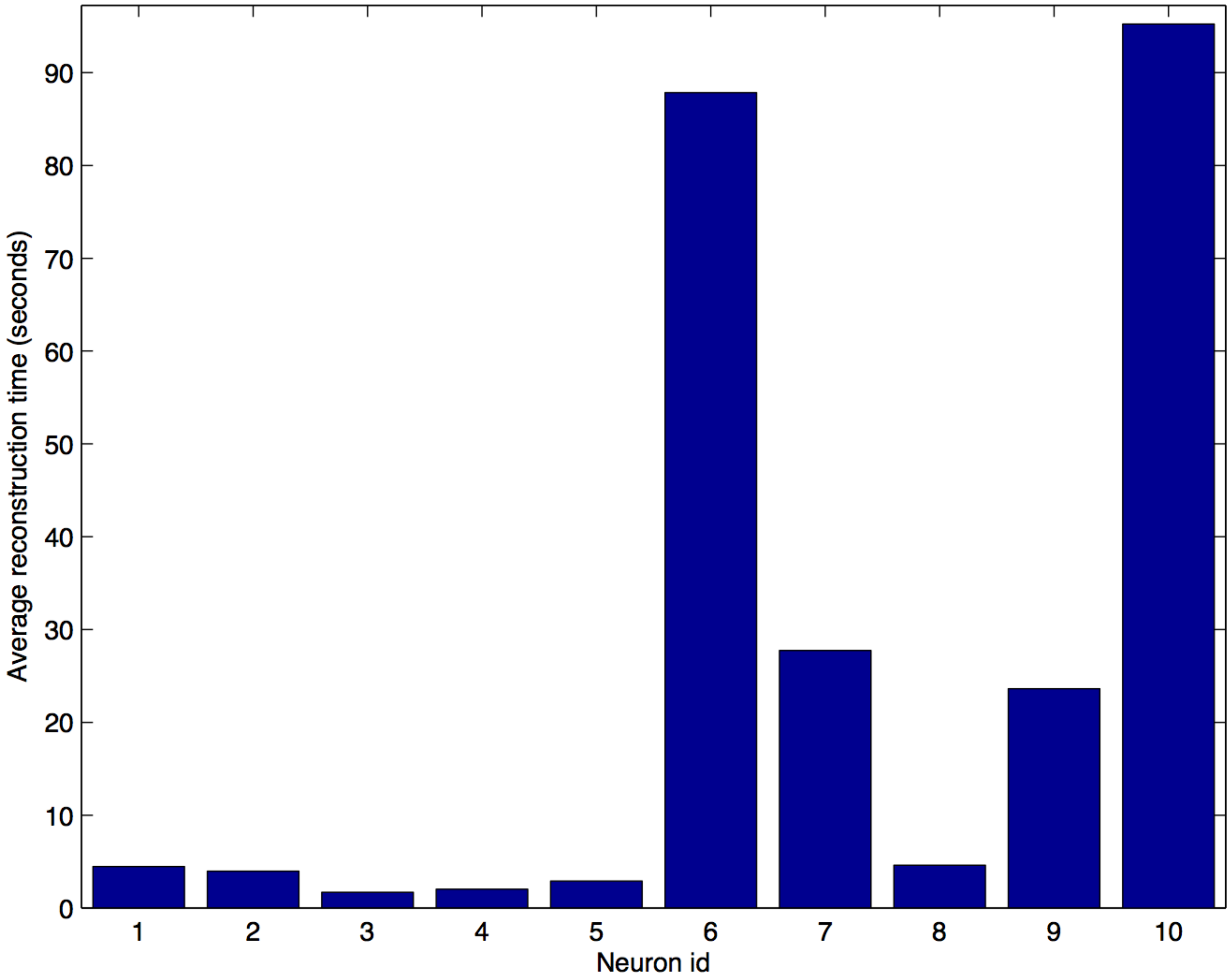

Supplementary Fig. 5. Vaa3D-Neuron2 reconstruction time of 10 *Drosophila* neurons.

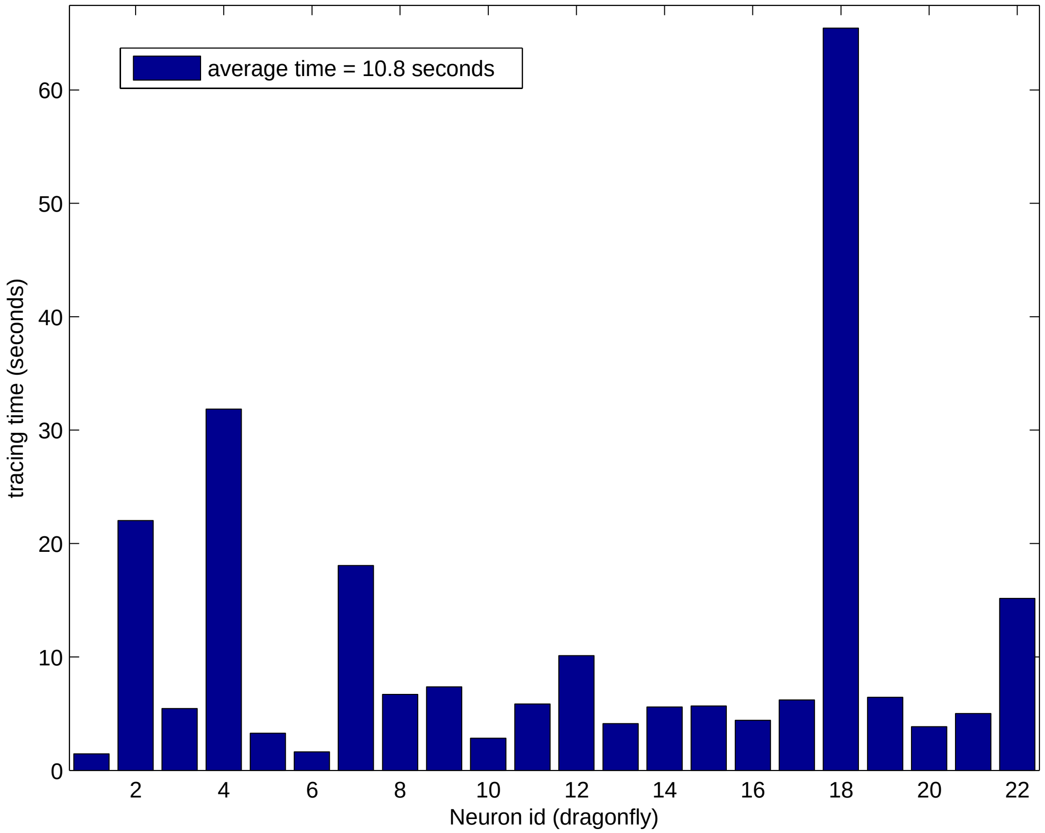

Supplementary Fig. 6. Vaa3D-Neuron2 reconstruction time of 22 dragonfly neurons.

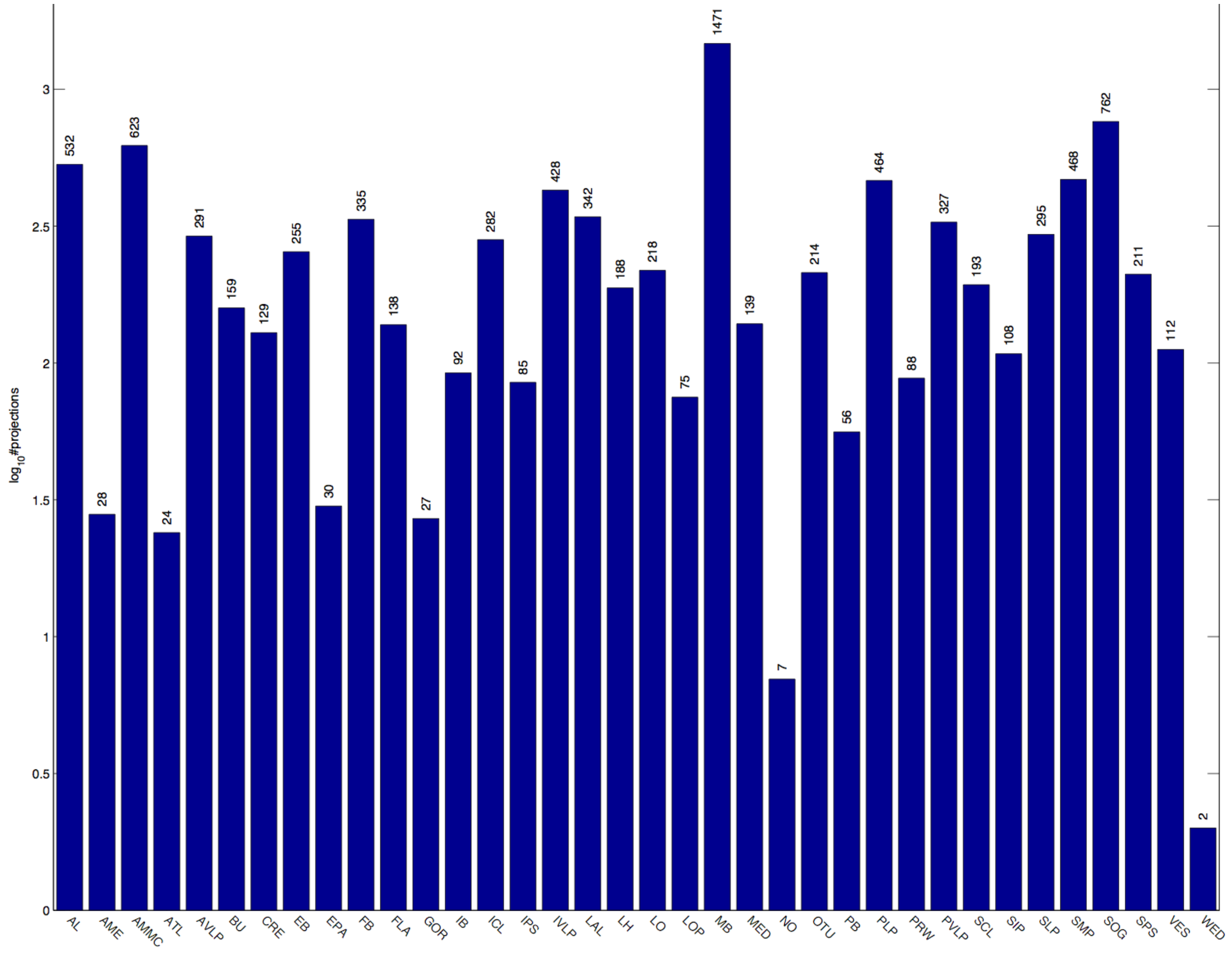

Supplementary Fig. 7. The number of reconstructed projections of each *Drosophila* brain compartment (see Fig. 7b for the complete names of these compartments). The total number is also shown on top of each bar.
